# Estimation of the cost-effectiveness of HIV prevention portfolios for people who inject drugs in the United States: A model-based analysis

**DOI:** 10.1371/journal.pmed.1002312

**Published:** 2017-05-24

**Authors:** Cora L. Bernard, Douglas K. Owens, Jeremy D. Goldhaber-Fiebert, Margaret L. Brandeau

**Affiliations:** 1 Department of Management Science and Engineering, Stanford University, Stanford, California, United States of America; 2 VA Palo Alto Health Care System, Palo Alto, California, United States of America; 3 Stanford Health Policy, Centers for Health Policy and Primary Care and Outcomes Research, Stanford University, Stanford, California, United States of America; Massachusetts General Hospital, UNITED STATES

## Abstract

**Background:**

The risks of HIV transmission associated with the opioid epidemic make cost-effective programs for people who inject drugs (PWID) a public health priority. Some of these programs have benefits beyond prevention of HIV—a critical consideration given that injection drug use is increasing across most United States demographic groups. To identify high-value HIV prevention program portfolios for US PWID, we consider combinations of four interventions with demonstrated efficacy: opioid agonist therapy (OAT), needle and syringe programs (NSPs), HIV testing and treatment (Test & Treat), and oral HIV pre-exposure prophylaxis (PrEP).

**Methods and findings:**

We adapted an empirically calibrated dynamic compartmental model and used it to assess the discounted costs (in 2015 US dollars), health outcomes (HIV infections averted, change in HIV prevalence, and discounted quality-adjusted life years [QALYs]), and incremental cost-effectiveness ratios (ICERs) of the four prevention programs, considered singly and in combination over a 20-y time horizon. We obtained epidemiologic, economic, and health utility parameter estimates from the literature, previously published models, and expert opinion. We estimate that expansions of OAT, NSPs, and Test & Treat implemented singly up to 50% coverage levels can be cost-effective relative to the next highest coverage level (low, medium, and high at 40%, 45%, and 50%, respectively) and that OAT, which we assume to have immediate and direct health benefits for the individual, has the potential to be the highest value investment, even under scenarios where it prevents fewer infections than other programs. Although a model-based analysis can provide only estimates of health outcomes, we project that, over 20 y, 50% coverage with OAT could avert up to 22,000 (95% CI: 5,200, 46,000) infections and cost US$18,000 (95% CI: US$14,000, US$24,000) per QALY gained, 50% NSP coverage could avert up to 35,000 (95% CI: 8,900, 43,000) infections and cost US$25,000 (95% CI: US$7,000, US$76,000) per QALY gained, 50% Test & Treat coverage could avert up to 6,700 (95% CI: 1,200, 16,000) infections and cost US$27,000 (95% CI: US$15,000, US$48,000) per QALY gained, and 50% PrEP coverage could avert up to 37,000 (22,000, 58,000) infections and cost US$300,000 (95% CI: US$162,000, US$667,000) per QALY gained. When coverage expansions are allowed to include combined investment with other programs and are compared to the next best intervention, the model projects that scaling OAT coverage up to 50%, then scaling NSP coverage to 50%, then scaling Test & Treat coverage to 50% can be cost-effective, with each coverage expansion having the potential to cost less than US$50,000 per QALY gained relative to the next best portfolio. In probabilistic sensitivity analyses, 59% of portfolios prioritized the addition of OAT and 41% prioritized the addition of NSPs, while PrEP was not likely to be a priority nor a cost-effective addition. Our findings are intended to be illustrative, as data on achievable coverage are limited and, in practice, the expansion scenarios considered may exceed feasible levels. We assumed independence of interventions and constant returns to scale. Extensive sensitivity analyses allowed us to assess parameter sensitivity, but the use of a dynamic compartmental model limited the exploration of structural sensitivities.

**Conclusions:**

We estimate that OAT, NSPs, and Test & Treat, implemented singly or in combination, have the potential to effectively and cost-effectively prevent HIV in US PWID. PrEP is not likely to be cost-effective in this population, based on the scenarios we evaluated. While local budgets or policy may constrain feasible coverage levels for the various interventions, our findings suggest that investments in combined prevention programs can substantially reduce HIV transmission and improve health outcomes among PWID.

## Abstract 2

In a modeling study, Cora Bernard and colleagues examine the cost-effectiveness of opioid agonist therapy (OAT), needle-syringe exchange programs (NSP), screening and intensive antiretroviral therapy programs (Test & Treat), and oral HIV pre-exposure prophylaxis (PrEP) for prevention of HIV in people who inject drugs.

## Author summary

**Why was this study done?:**

- The US drug-injecting population is growing, with consequent deaths from opioid overdose (30,000 deaths in 2014 alone) as well as increased HIV transmission.
- Cost-effective HIV prevention programs for people who inject drugs are essential to the long-term health outcomes for this population and other high-risk groups in the US.
- Our model-based analysis was designed to identify high-value portfolios of HIV prevention programs targeted to people who inject drugs in the US.

**What did the researchers do and find?:**

- We adapted and extended a dynamic compartmental model that tracks multiple HIV risk groups in the US.
- We projected the lifetime costs and benefits resulting from combinations of opioid agonist therapy, needle and syringe exchange programs, enhanced HIV screening and antiretroviral therapy programs, and oral HIV pre-exposure prophylaxis (PrEP) for people who inject drugs.
- We estimated that opioid agonist therapy, which reduces injecting frequency and results in multiple, immediate quality-of-life improvements, can be cost-effective, while less expensive programs like needle and syringe exchange can provide additional value when used in combination. PrEP was not likely to be cost-effective in this population in most of the scenarios we evaluated.

**What do these findings mean?:**

- Several prevention interventions—opioid agonist therapy, needle and syringe exchange programs, and enhanced HIV testing and treatment services—have the potential to prevent HIV transmission in PWID and improve health outcomes while meeting commonly accepted thresholds for cost-effectiveness. Opioid agonist therapy may reduce HIV transmission and also improve quality of life for people who inject drugs; both effects contribute to its favorable cost-effectiveness.
- Some prevention programs for people who inject drugs are controversial, and access is limited. We demonstrate that some of these programs can be both effective and cost-effective investments that have the potential to reduce not only the spread of HIV but also the size of the injecting population.

## Introduction

Over the past decade, injection drug use, particularly heroin injection, has increased across most US demographic groups, making substance-abuse-related mortality and morbidity a public health crisis [1]. In 2014, there were 47,055 deaths from drug overdose in the US, with almost 30,000 due to opioid overdose [2]. Because HIV spreads relatively efficiently through the transfer of blood in shared injecting equipment [3], people who inject drugs (PWID) account for a disproportionate share of HIV prevalence and incidence in the US [4,5]. Although HIV prevalence and incidence among US PWID have been falling over the past decade [4,6,7], recent growth in the size of the injecting population has raised concerns that HIV risks could rise [8]. Programs targeted to PWID, which have the additional benefit of preventing downstream sexual transmission of HIV to others in the population, are therefore a public health priority. Given the current epidemic of injection drug use in the US, benefits that extend beyond HIV prevention are also a critical consideration [1,8].

A recent empirical study demonstrated that a combined prevention strategy effectively halted HIV epidemics in PWID populations [9]. This strategy included opioid agonist therapy (OAT), which reduces injecting frequency [10,11], needle and syringe programs (NSPs), which reduce injection equipment sharing [12], and enhanced services for HIV testing and treatment (Test & Treat), which identify and virally suppress infected individuals by enrolling them in antiretroviral therapy (ART) [13,14]. Additionally, the US Centers for Disease Control and Prevention (CDC) now recommends daily oral pre-exposure prophylaxis (PrEP), which reduces uninfected individuals’ risk of acquiring HIV, for PWID [15]. Although all of these programs have demonstrated efficacy, they have diverse delivery methods and target populations, as well as different costs and anticipated benefits, and have not been modeled comparatively in a cost-effectiveness context.

The complexity of transmission dynamics and intervention scenarios makes it difficult to deduce a priori the highest value portfolio of prevention programs for PWID. To address this, we extended an empirically calibrated model of the US HIV epidemic [16] to assess the cost-effectiveness of alternative HIV prevention portfolios for US PWID. Each portfolio included some combination of OAT, NSPs, Test & Treat, and PrEP scaled to various coverage levels. Our model integrated clinical, epidemiologic, and economic data and captured the dynamic accrual of total population costs and benefits by tracking the spread of HIV through injection-based and sexual transmission routes.

## Methods

### Overview

**Fig 1:**
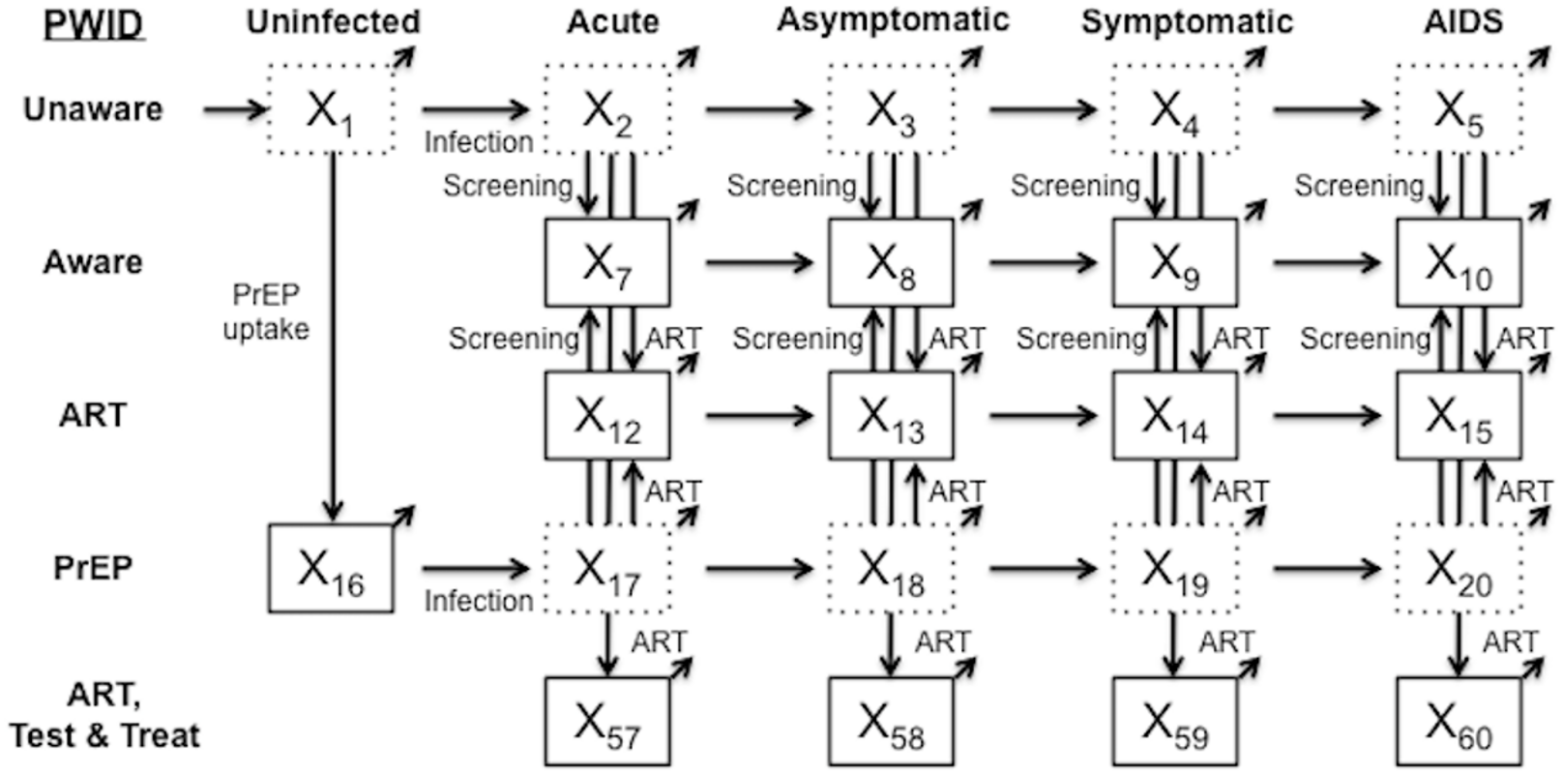
Simplified model schematic. The simplified schematic shows model compartments for PWID not on OAT. A parallel compartment set exists for PWID on OAT, with flows between the two compartment sets, as well as 13 compartments each for men who have sex with men and low-risk populations, yielding a total of 70 active compartments in the model. Individuals age into the model uninfected at age 18 y (external arrow into compartment X_1_) and can exit from any compartment (diagonal arrows) due to maturation out of the modeled population (at age 65 y) or death. Solid compartment borders denote awareness of HIV status. ART, antiretroviral therapy; OAT, opioid agonist therapy; PrEP, pre-exposure prophylaxis; PWID, people who inject drugs; Test & Treat, HIV testing and treatment.

Our analysis builds on a previously published dynamic compartmental model of the US HIV epidemic [16]. A simplified schematic (Fig 1) illustrates how the model stratifies the adult population aged 18–64 y by HIV infection and awareness status, CD4 count, ART status, OAT status, and risk group. We instantiated the model with US data (Table 1) and calibrated it to match a range of targets, including CDC estimates of US HIV prevalence [5,17–21] and incidence [4,21] across all risk groups. The model consists of a system of differential equations programmed in Matlab R2015b (MathWorks) that track compartment populations monthly from 2015 to 2035. We used a societal perspective to calculate the costs, quality-adjusted life years (QALYs), and incremental cost-effectiveness ratios (ICERs) associated with each portfolio of interventions. Costs and QALYs were measured over the lifetimes of all individuals active in the model over the 20-y time horizon and were discounted at 3% annually [22,23]. We also measured health outcomes such as HIV infections averted and change in HIV prevalence.

**Table 1:** Estimates for key model parameters.

Parameter	Value	Range*	Source
**Demographics**			
PWID population (18–64 y)	1.1 million	0.8–1.4 million	[24–26]
HIV prevalence, PWID	9.8%	8.0%–12.1%	[18]
**OAT**			
Initial PWID enrollment	24.8%	13.2%–34.4%	[17,18,27–29]
Percent of PWID quitting OAT annually	32.1%	18.1%–43.6%	[28,30]
Percent of PWID quitting drug use annually	3.6%	1.9%–5.4%	[28,30]
Percent decrease in risky injections	54.7%	0.004%–82.2%^†^	[10,11]
Mortality hazard ratio	0.39	0.12–0.96	[27,31]
Quality-of-life multiplier	1.06	[1.00, 1.06]	[27,28,32]
Start-up cost^‡^	700	[350, 1,400]	Estimated [27,28,33]
Annual cost^§^	7,000	[4,320, 10,430]	[27,28,33]
**NSPs**			
Percent decrease in shared equipment	45%	[20%, 80%]	[12,34]
Start-up cost^‡^	0	[0, 3,600]	[27,28,35–37]
Annual cost^§^	615	[308, 1,230]	[35–37]
**Test & Treat**			
Initial percent of PWID aware of HIV status	69.9%	59.1%–78.6%	[17,18]
Decrease in risky injections due to awareness of HIV status	23.2%	0%–55.0%^†^	[27,29]
Relative risk of condom use due to awareness of HIV status	1.66	1.21–2.17	[18,27,29,38]
Initial PWID enrollment in ART	40.9%	29.3%–51.7%	[39,40]
Increase in life expectancy on ART, years	16.5	10.3–29.0	[41–43]
Transmission reduction if injecting partner is on ART	59%	14%–82%	[13,44]
Transmission reduction if sexual partner is on ART	90%	68%–99%	[14,41,44]
Percent of PWID entering community-based care program following HIV diagnosis	75%	[50%, 90%]	Estimated [39,40,45,46]
Percent of PWID in community-based care program who remain on ART after maturing from model	100%	[30%, 100%]	Estimated [39,40,45,46]
Percent of PWID in community-based care program who remain on ART at end of intervention	75%	[30%, 100%]	Estimated [39,40,45,46]
Quality-of-life multiplier (ART and community-based care program)	1.18	[1.15, 1.22]	[32,44,47–49]
Annual cost of ART^§^	23,300	[13,700, 35,500]	[44,47,50,51]
Annual cost of community-based care program^§^	6,600	[3,300, 13,200]	[49]
One-time cost of positive HIV diagnosis	500	110–1,210	[48]
One-time cost of negative HIV diagnosis	50	20–100	[48]
**PrEP**			
Injection-based and sexual transmission reduction	48.9%	10.0%–89.1%	[13]
Screening frequency, months	3.0	1.2–4.9	[15]
Percent immediately initiating ART following HIV diagnosis	50%	10%–90%	Estimated [39,40,45,46]
Annual cost of drug^§^	10,000	[1,000, 14,000]	[48,52–54]
Annual cost of screening services^§^	800	100, 2,240	[48,51]

*Bracketed tuples refer to the range explored in sensitivity analyses for intervention parameters.

^†^S1 Appendix, Section 5.3, addresses the motivation behind and implications of wide ranges.

^‡^Approximate cost per additional person covered by intervention, in 2015 US dollars.

^§^Cost per person, in 2015 US dollars.

ART, antiretroviral therapy; NSP, needle and syringe program; OAT, opioid agonist therapy; PrEP, pre-exposure prophylaxis; PWID, people who inject drugs; Test & Treat, HIV testing and treatment.

### Model structure and flows

The majority of the modeled population is considered low-risk heterosexual. Consistent with CDC estimates, PWID and men who have sex with men (MSM) are smaller populations with higher initial HIV prevalence and HIV-related risk behaviors [5,21]. Between 2015 and 2035, US birth cohorts age into the model when they turn 18 y, and individuals either age out of the actively injecting population at age 65 y or die (at age <65 y) at background mortality rates adjusted for risk behavior [31,48,55], HIV infection, and ART status [39,40,42,43,47]. Additional Markov models follow individuals who mature out and those alive at the end of the 20-y analytic time horizon to capture all lifetime costs and benefits (S1 Appendix, Section 1). The model tracks incident infections, disease progression, HIV screening, enrollment in ART, and transitions into and out of OAT.

Through calibration, the model reflects HIV infection risks given current OAT, NSP, Test & Treat, and PrEP coverage. We assume that PWID, when they do share injecting equipment, are equally likely to share with any other PWID [17,27,28,38,44]. Injection-based HIV transmission depends on the infected partner’s HIV stage [3,41] and ART status [13,14], along with the uninfected partner’s use of PrEP [13]. Sexual mixing patterns approximate partnerships among and between risk groups, with transmission between sero-discordant partners additionally depending on male condom use [18–20,27,29,38,56], condom effectiveness [57], and whether both partners are MSM [3].

Upon infection, an individual enters a brief but highly infectious acute stage [3,41,58], followed by asymptomatic HIV (CD4 count 500 to 1,200 cells/mm^3^), symptomatic HIV (CD4 count >200 to <500 cells/mm^3^), and AIDS (CD4 count ≤ 200 cells/mm^3^) [25,41,47]. As CD4 count falls, infectivity increases [3,41,58] and quality of life decreases [44,59]. ART moderates these effects, reducing injection-based transmission by 59% [13,44] and sexual transmission by 90% [14,41,44], and extends life expectancy by suppressing HIV viral load [40,42,43]. To be eligible for ART, an individual must first be diagnosed with HIV infection, at which time that person may also modify risk behaviors, such as injection equipment sharing or condom usage [18,27,29,38]. In the model, HIV detection rates depend on risk group and are higher in symptomatic HIV compartments [47].

Although the model captures multiple risk groups in order to calibrate to the US HIV epidemic, all interventions in this analysis are directed exclusively to PWID. Program scale-ups are incremental to the status quo, which assumes baseline 2015 levels of OAT, NSP, Test & Treat, and PrEP coverage. Scale-ups to low, medium, and high coverage levels were chosen to standardize comparisons and provide intuition for program costs, benefits, and interactions as coverage increases. In practice, feasible enrollment levels in terms of budget and participant retention are likely to vary by community.

### Opioid agonist therapy

Methadone and buprenorphine are the most common pharmacological therapies prescribed as OAT in the US [10]. We assume that OAT decreases the number of injections by 55% [10,11], thereby reducing overdose risk [27,31] and the chance of HIV transmission, and improving quality of life [27,28,32]. Additionally, we assume that individuals on OAT have higher HIV screening rates than the general PWID population and are more likely to connect to ART services if diagnosed [60,61]. OAT also provides the sole pathway by which individuals permanently cease drug use (3.6% annually) and move to a lower-risk population [28,30]. Previous analyses of the costs and benefits of OAT have consistently found it to be cost-effective [28,62,63].

We assume that 25% of PWID receive OAT under the status quo [17,18,27–29]. At low, medium, and high coverage levels, enrollment increases to 40%, 45%, and 50% of the PWID population, respectively. Such scale-up would involve both short-term investments (e.g., overhead costs for starting methadone clinics) and the long-term costs of delivering the therapies themselves [27,28,33]. In sensitivity analyses, we varied parameters affecting OAT’s effectiveness and cost.

### Needle and syringe programs

In the model, NSP broadly refers to any of a range of local programs, such as those at pharmacies, hospitals, or designated facilities, through which PWID access sterile hypodermic injecting equipment [64]. We assume that NSPs reduce equipment sharing by 45% [12,34]. Despite being considered a cost-effective HIV prevention strategy [35,65], social and political barriers often prevent NSP expansion [66], and it remains the most controversial of PWID-targeted interventions.

We calibrated our analysis to the current effects of NSPs, which we assume to be minimal at a national level in the status quo [37,67]. Low, medium, and high coverage levels expand NSPs to reach 40%, 45%, and 50% of PWID, respectively. We assume a fixed annual operating budget for NSPs, and low scale-up costs [35–37]. We varied the estimated costs and effectiveness of NSPs in sensitivity analyses.

### HIV testing and treatment

US guidelines recently eliminated a CD4 count threshold for ART initiation and now recommend immediate ART following diagnosis [68]. US cities adopting this policy have used aggressive HIV testing, same-day treatment initiation, and routine follow-up to significantly increase overall viral suppression [69,70]. Previous analyses of Test & Treat in the US have found favorable cost-effectiveness ratios [44,47,71], and while PWID remain a difficult demographic to reach and sustain in care [70], other community-based interventions targeted exclusively to high-risk populations have demonstrated the potential for sustained case management for PWID [49].

Our status quo reflects current ART engagement levels [27,39,40], with risk group determining the probability that a newly diagnosed individual becomes virally suppressed [40,72]. At low, medium, and high coverage levels of Test & Treat, 40%, 45%, and 50% of infected PWID, respectively, enroll in sustained ART care by 2035. (Because an infection must occur before enrollment in Test & Treat, this intervention, unlike the others, cannot scale immediately in the model.) Costs associated with Test & Treat include those of screening, diagnosis confirmation, counseling, and ART [44,47,48,50,51], as well as the associated costs of comprehensive community-based care programs [49]. We varied the parameters determining enrollment probability (calibrated to additionally reflect the phenomenon of loss to follow-up), program costs, and participant quality-of-life benefits in sensitivity analyses.

### Pre-exposure prophylaxis

Our previous analysis [16] found that PrEP, a daily oral pill of 300 mg tenofovir disoproxil fumarate and 200 mg emtricitabine (Truvada), is most valuable for PWID when delivered per the CDC’s clinical guidelines (e.g., HIV screening every 3 mo, toxicity monitoring every 6 mo) [15] and with prompt and sustained provision of ART for those who do become infected. Nonetheless, PrEP for PWID is expensive in terms of total budget outlay and ICER, although it could be considered cost-effective in the highest prevalence communities [16].

Our status quo assumes negligible PrEP use among PWID in 2015. At low, medium, and high coverage levels, 40%, 45%, and 50% of uninfected PWID, respectively, receive PrEP. The direct costs of PrEP reflect the costs of Truvada [48,53,54] as well as ongoing monitoring costs [48,51]. We assume that the PrEP enrollment process modestly increases HIV screening for the entire PWID population and that individuals diagnosed with HIV discontinue PrEP immediately [15].

### Economic model

Each compartment is associated with an annual cost (adjusted for inflation to 2015 US dollars [73]) and QALY value depending on the characteristics of that subpopulation. A one-time scale-up cost is additionally associated with each intervention (S1 Appendix, Section 3). For every individual in the model, discounted costs and QALYs accrue at each time step, yielding a total cost and QALY estimate for each scenario. We also include lifetime costs and QALYs for individuals maturing out of the population and for individuals alive in the population at the end of the modeled time horizon (S1 Appendix, Section 1). We calculate ICERs by comparing the incremental discounted costs and QALYs for each scenario to the next best alternative [23].

### Model calibration

We used a random search algorithm to repeatedly sample from estimated distributions for each model input and then empirically fit the model to US epidemiologic data, resulting in 182 calibrated parameter sets (S1 Appendix, Section 2) [74,75]. Ranges on parameter values are presented in Table 1. We performed all analyses over the calibrated sets to incorporate parameter uncertainty [76]. We present the averages over these sets as our base case results, with 95% confidence intervals where appropriate.

## Results

### Main analysis

Table 2 compares the four prevention programs implemented singly. We estimate that expansions of OAT, NSPs, and Test & Treat up to coverage levels of 50% can cost less than US$30,000 per QALY gained relative to the next highest coverage level (95% confidence intervals for these programs fall below commonly accepted thresholds of cost-effectiveness, with NSPs having the widest range), while PrEP is likely to cost more than US$300,000 per QALY gained (95% confidence intervals fall above commonly accepted thresholds of cost-effectiveness). Our model estimates that achieving 50% coverage for OAT may avert fewer infections than achieving 50% coverage for PrEP. Because we model nearly immediate direct decreases in mortality rates and increases in quality of life and HIV treatment for those enrolled in OAT, we estimate that expanded OAT coverage is likely to produce higher QALY gains than any other intervention, even when averting fewer infections. An underlying assumption in the model is that PWID must transition through OAT before being “eligible” to cease injection use. At low, medium, and high OAT coverage, respectively, we estimate that the actively injecting population could decrease by 23%, 31%, and 37% over 20 y, accounting for a substantial proportion of the quality-of-life gains. Relatively low delivery costs combined with these benefits have the potential to make OAT the most cost-effective choice among the singly implemented prevention programs.

**Table 2:** Cost-effectiveness of interventions considered singly.

Intervention	Additional PWID covered per month*	PWID HIV infections averted (thousands)^†^	Percent change in PWID HIV prevalence at 20 y^†^	Total costs (US dollars, billions)	Total QALYs (billions)	Incremental costs (US dollars, billions)	Incremental QALYs (thousands)	ICER (US dollars, thousands)^†^
**Status quo**	*—*	*—*	*—*	32,528	6.4340	*—*	*—*	—
**OAT**								
Low coverage	14.0%	14 (2.8, 30)	−10 (−2.2, −22)	32,552	6.4353	24.0	1,340	18 (14, 24)
Medium coverage	19.0%	18 (4.4, 37)	−13 (−3.1, −27)	32,559	6.4357	7.4	413	18 (14, 24)
High coverage	24.0%	22 (5.2, 46)	−16 (−3.5, −31)	32,567	6.4361	7.4	417	18 (14, 24)
**NSPs**								
Low coverage	40.0%	21 (7.1, 35)	−14 (−5.6, −22)	32,531	6.4341	2.8	141	20 (6, 74)
Medium coverage	45.0%	23 (8.0, 39)	−16 (−6.3, −25)	32,531	6.4341	0.4	16	25 (7, 80)
High coverage	50.0%	35 (8.9, 43)	−17 (−7.0, −27)	32,531	6.4342	0.4	16	25 (7, 76)
**Test & Treat**								
Low coverage	0.2%	1.3 (0.04, 3.4)	0 (−1.2, 1.1)	32,528	6.4340	0.7	25	28 (16, 45)
Medium coverage	0.7%	3.5 (0.75, 8.8)	0 (−3.2, 2.9)	32,531	6.4340	1.0	37	27 (15, 51)
High coverage	1.2%	6.7 (1.2, 16)	0 (−5.7, 4.1)	32,531	6.4341	1.5	56	27 (15, 48)
**PrEP**								
Low coverage	36.0%	31(18, 49)	−21 (−14, −30)	32,597	6.4342	69.1	220	314 (162, 667)
Medium coverage	40.5%	34 (20, 54)	−24 (−16, −33)	32,606	6.4342	8.8	25	352 (189, 713)
High coverage	45.0%	37 (22, 58)	−26 (−17, 36)	32,615	6.4343	8.8	24	367 (196, 684)

Each intervention is considered in isolation, with costs and QALYs for low coverage compared to the status quo, medium compared to low, and high compared to medium. Infections averted and change in prevalence are directly compared to the status quo. Low, medium, and high coverage correspond to 40%, 45%, and 50% enrollment of the eligible population, respectively.

*Compared to status quo and expressed as percent of total PWID population. Note that interventions are defined by expanding *up to* a standardized coverage level within the *eligible* population, which varies substantially between programs.

^†^Mean and 95% confidence interval over calibrated sets.

ICER, incremental cost-effectiveness ratio; NSP, needle and syringe program; OAT, opioid agonist therapy; PrEP, pre-exposure prophylaxis; PWID, people who inject drugs; QALY, quality-adjusted life year; Test & Treat, HIV testing and treatment.

**Fig 2:**
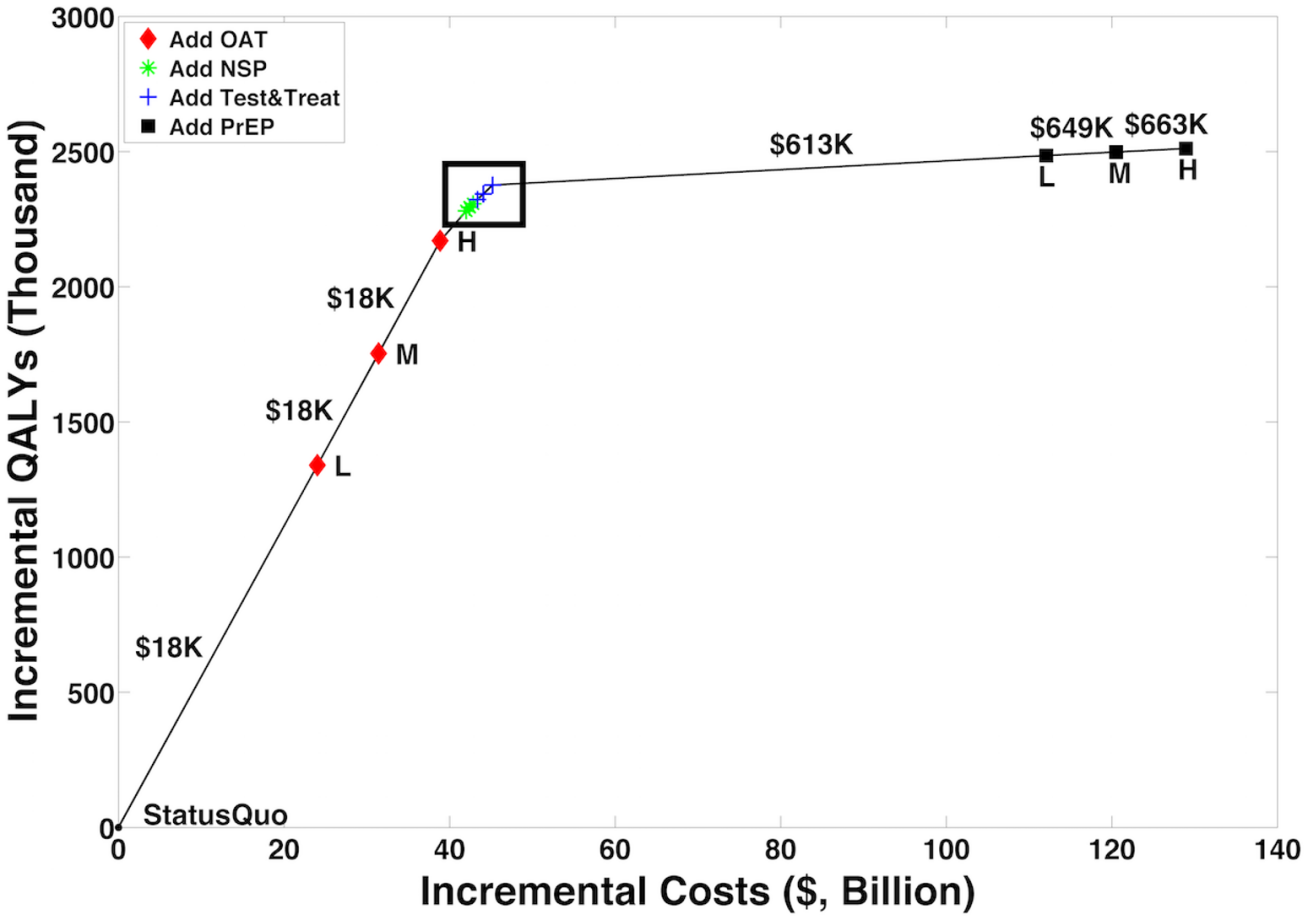
Cost-efficient frontier. We considered combinations of OAT, NSPs, Test & Treat, and PrEP at status quo, low (L), medium (M), and high (H) coverage levels and plotted the resulting cost-efficient frontier, with incremental QALYs on the *y*-axis versus incremental costs on the *x*-axis. Fig 3 is an enlargement of the region indicated by the box. The figure illustrates our model’s projections that prioritizing expansions of OAT coverage and then investing in NSPs and Test & Treat can deliver a high-value portfolio of interventions. OAT, opioid agonist therapy; NSP, needle and syringe program; PrEP, pre-exposure prophylaxis; QALY, quality-adjusted life year; Test & Treat, HIV testing and treatment.

**Fig 3:**
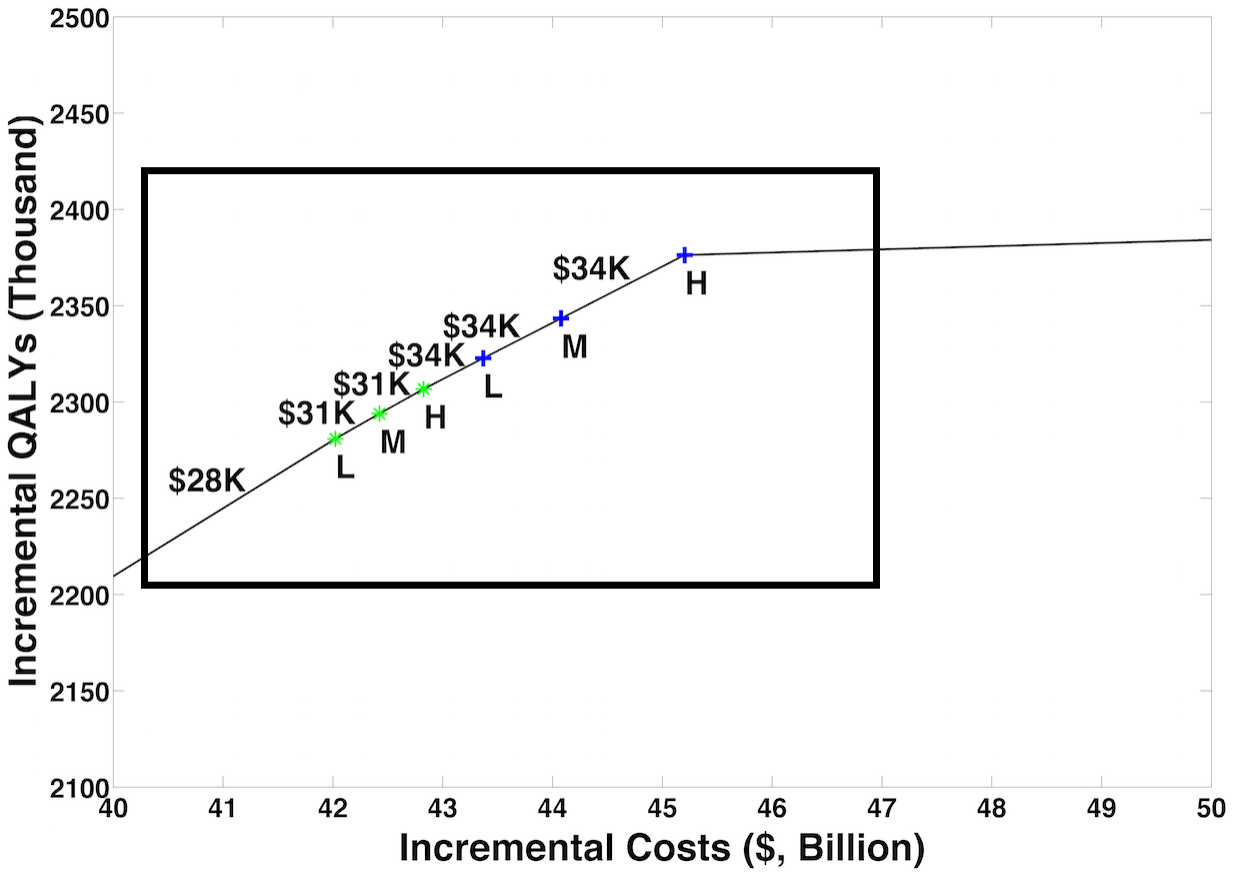
Cost-efficient frontier—Enlargement. Fig 3 is an enlargement of the region indicated by the box in Fig 2. Symbols as in Fig 2. L, low; M, medium; H, high; QALY, quality-adjusted life year.

Figs 2 and 3 and Table 3 illustrate how programs can be combined to construct the highest value prevention portfolio from all considered combinations of OAT, NSPs, Test & Treat, and PrEP. Our base case analysis indicates that scaling OAT coverage up to 50%, then scaling NSP coverage to 50%, then scaling Test & Treat coverage to 50% can be a cost-effective approach to maximizing health benefit, with each additional coverage expansion having the potential to cost less than US$50,000 per QALY gained relative to the next best portfolio. Over 20 y, the combination of high OAT, NSP, and Test & Treat coverage can avert up to 43,400 (95% CI: 23,000, 74,000) infections and decrease HIV prevalence among PWID by 27% (95% CI: 12%, 45%). This is 5,700 more infections and a 1% greater decrease in prevalence than model projections from a program of high PrEP alone. At the same time, our analysis estimates that combinations of OAT, NSPs, and Test & Treat could cost up to US$40 billion less than a high-coverage PrEP program over 20 y.

**Table 3:** Prevention portfolios on the cost-efficient frontier.

Intervention Portfolio	PWID HIV infections averted (thousands)*	Percent change in PWID HIV prevalence at 20 y*	Total costs (US dollars, billions)	Total QALYs (billions)	Incremental costs (US dollars, billions)	Incremental QALYs (thousands)	ICER (US dollars)^†^
Status quo	—	—	32,528	6.4340	—	—	—
Low OAT	14 (2.8, 30)	−10 (−2.2, −22)	32,552	6.4353	24.0	1,340	18,000
Medium OAT	18 (4.4, 37)	−13 (−3.1, −27)	32,559	6.4357	7.4	413	18,000
High OAT	22 (5.2, 46)	−16 (−3.5, −31)	32,566	6.4361	7.4	417	18,000
High OAT, low NSP	37 (16, 68)	−26 (−11, −43)	32,569	6.4362	3.1	111	28,000
High OAT, medium NSP	38 (16, 70)	−27 (−12, −44)	32,569	6.4362	0.4	13	31,000
High OAT, high NSP	40 (17, 72)	−28 (−13, −45)	32,569	6.4362	0.4	13	31,000
High OAT, high NSP, low Test & Treat	41 (20, 72)	−28 (−12, −45)	32,570	6.4362	0.5	16	34,000
High OAT, high NSP, medium Test & Treat	42 (21, 74)	−28 (−13, −45)	32,571	6.4362	0.7	21	34,000
High OAT, high NSP, high Test & Treat	43 (23, 74)	−27 (−12, −45)	32,572	6.4362	1.1	33	34,000
High OAT, high NSP, high Test & Treat, low PrEP	59 (35, 89)	−38 (−25, −54)	32,639	6.4363	66.9	109	613,000
High OAT, high NSP, high Test & Treat, medium PrEP	61 (36, 91)	−40 (−25, −55)	32,647	6.4363	8.4	13	649,000
High OAT, high NSP, high Test & Treat, high PrEP	62 (37, 92)	−41 (−26, −56)	32,655	6.4363	8.4	13	663,000

Incremental costs, incremental QALYs, and the ICER of each portfolio are relative to the next best intervention. Infections averted and change in prevalence are directly compared to the status quo.

*Mean and 95% confidence interval over calibrated sets.

^†^ICER uncertainty under one-way sensitivity analysis is explored in S1 Appendix, Sections 5.1 and 5.3, and Table A in S1 Appendix, and under probabilistic sensitivity analysis, in S1 Appendix, Section 5.4, and Table D1 in S1 Appendix.

ICER, incremental cost-effectiveness ratio; NSP, needle and syringe program; OAT, opioid agonist therapy; PrEP, pre-exposure prophylaxis; PWID, people who inject drugs; QALY, quality-adjusted life year; Test & Treat, HIV testing and treatment.

### Sensitivity analysis

We conducted multiple sensitivity analyses to assess the importance of uncertainty around model parameters and to evaluate factors important to developing high-value portfolios. We present key insights below, with further details in S1 Appendix, Section 5.

Our findings presented in Figs 2 and 3 and Table 3 are largely insensitive to the majority of one-way sensitivity analyses conducted on model parameters relating to the implementation of each intervention. Several one-way sensitivity analyses conducted on program delivery parameters cause an intuitive interchange in the relative priority of interventions. For instance, at low NSP delivery costs or high efficacy of NSPs, or at high OAT delivery and start-up costs, our analysis suggests that the highest value portfolio would first increase NSP coverage before scaling up OAT. In probabilistic sensitivity analysis (PSA), 41% of sampled sets first add NSPs to the portfolio before adding OAT, while the rest add OAT first. We estimate that additions of OAT and NSPs to the portfolio cost less than US$50,000 per QALY gained in 100% and 74% of all samples considered in PSA, respectively. When the threshold is US$100,000 per QALY gained, NSPs are a cost-effective addition in 93% of PSA samples.

We estimate that increasing Test & Treat coverage could cost less than US$50,000 per QALY gained over a range of delivery costs. We did not identify a one-way sensitivity scenario under which Test & Treat replaces OAT as the most favorable investment, although there are several scenarios in which, unlike in the base case, Test & Treat is a higher priority investment than NSPs. Furthermore, in PSA there were no sampled sets for which the model projected Test & Treat as the first addition to a highest value portfolio. The addition of Test & Treat costs less than US$50,000 per QALY gained in 4.7% of PSA samples, less than US$100,000 in 33% of samples, and less than US$150,000 in 67% of samples. Thus, in terms of cost-effectiveness, our findings indicate that a portfolio of prevention programs could achieve highest value by first investing in OAT or NSPs before scaling up Test & Treat.

Over the majority of one-way sensitivity analyses, we estimate PrEP to cost more than US$500,000 per QALY gained when added to the portfolio. Only when we decrease PrEP’s drug cost by 90% do we project its ICER value to fall below US$100,000 relative to the next best alternative. In PSA, when we vary both the cost and the efficacy of PrEP within estimated, currently feasible ranges, 1% of sampled sets have a highest value portfolio for which the addition of PrEP costs less than US$150,000 per QALY gained.

To further explore the dependence of our results on underlying calibrated parameters, such as ART efficacy, we performed all analyses on a limited subset, the sets containing a parameter value in the bottom or top 5% of all sets, for each parameter. Limiting the analysis to extreme values does not substantially change our findings, nor do other sensitivity analyses on the duration or implementation of interventions (S1 Appendix, Section 5.2). To probe the effects of wide confidence intervals on several parameters, we performed multiple joint sensitivity analyses outside the calibrated context as well as over three calibrated sets for which both OAT’s effectiveness in reducing injection frequency and the decrease in injection equipment sharing following HIV diagnosis (with implications for the effectiveness of Test & Treat) were highly unfavorable (S1 Appendix, Section 5.3). We estimated that in such circumstances NSPs can replace OAT as the priority investment, but OAT, NSPs, and Test & Treat remain cost-effective additions to the portfolio, while PrEP is not likely to be, although its value can increase when other interventions are less favorable.

## Discussion

The opioid epidemic is a global public health burden that has become particularly acute in the US [1,2,8]. In addition to the substantial mortality associated with substance abuse [2], high rates of HIV transmission among PWID make the successful prevention of HIV in this population a public health priority. To that end, we consider portfolios of HIV prevention programs that include OAT, NSPs, Test & Treat, and PrEP scaled to various coverage levels. Although model projections can only provide estimates of health benefits and costs, such analyses can provide intuition around critical mechanisms and assumptions to inform decision making. Our main finding is that, over 20 y, high coverage (enrollment of 50% of the eligible population) of OAT, NSPs, and Test & Treat in combination could avert nearly 43,400 (95% CI: 23,000, 74,000) HIV infections among PWID and reduce HIV prevalence among PWID by 27% (95% CI: 12%, 45%). The construction of such a portfolio has the potential to be cost-effective at each incremental expansion, with projected ICERs below US$50,000 per QALY gained. Moreover, our analysis suggests that the estimated benefit obtainable by PrEP alone (measured in QALYs) could potentially be achieved and even surpassed at substantially lower cost by combining other prevention interventions into high-value portfolios.

Advocates for efficient investment in PWID-specific interventions have asked, “What good is preventing HIV if we do not first save that life at HIV risk?” [77]. Our analysis suggests that the high competing mortality risks of PWID can explain why interventions that immediately improve quality of life can have substantially higher estimated benefits than those that focus on HIV prevention alone. Our analysis estimates that OAT, in particular, which we assume has a direct impact on the length and quality of life of treated individuals [27,28,30–32,60,61], can provide substantially more benefit, measured in QALYs, than other interventions, even when it prevents fewer infections (Table 2).

Although our analysis did not identify a scenario in which OAT was not a cost-effective addition to a high-value portfolio, deterministic and probabilistic sensitivity analyses can provide intuition regarding scenarios in which NSPs could replace OAT as the priority investment. Because the assumed delivery cost of NSPs is so much lower than that of other programs, our findings suggest that it is reasonable to invest in NSPs concurrent with OAT scale-up. While Test & Treat is often estimated in our analysis to be a cost-effective addition to the portfolio, our model does not project it to be a priority investment. Our estimates for ART’s reduction of transmission risk via injection-based contact [13,44] are lower than those for sexual contact [14,41,44], which may explain our projection of smaller benefits in the PWID population. It should also be noted that HIV prevalence in US PWID is less than 10% [18], and the direct QALY increases from Test & Treat programs were therefore low relative to programs that served the entire PWID population.

Costs and cost-effectiveness are but one factor among several in the decision to provide prevention interventions. Policymakers and clinicians may decide that considerations of ethics and social justice outweigh economic considerations for this vulnerable population. PrEP, for instance, can provide benefits for PWID and should not be denied on the basis of injection drug use. Moreover, our findings are based on the current cost of PrEP in the US. If the cost of PrEP were substantially reduced, its cost-effectiveness could become more favorable. Nonetheless, as policymakers address the broader epidemic, our findings suggest that increasing the availability of a full spectrum of prevention interventions would have the highest health and economic benefits.

Our analysis assumes that each intervention is an available option, which is not true in many settings. Treatment and prevention programs for PWID remain controversial, and interventions may be infeasible for reasons beyond budgetary impact. Despite evidence of effectiveness and cost-effectiveness, a current ban in the US prevents federal funding for NSPs [67], and although a previous study found no significant correlation between neighborhood crime and treatment centers [78], proposals for new methadone clinics often face community opposition. Moreover, nearly 60% of individuals on methadone in the US receive insufficient dosing [79]. For this reason, the model’s calibrated sets reflected a wide range of possible effectiveness levels of methadone. If barriers to OAT access were lowered and treatment offered at international evidence-based standards, our analysis suggests that the value of this already cost-effective intervention could increase.

Our analysis has several limitations (Table E in S1 Appendix). First, although our model captures dynamic interactions between programs, we implement combinations of programs as independent. This means that the efficacy and cost of each program does not depend on the efficacy, cost, and impact of other programs at the individual level (for a PWID enrolled in multiple programs) or the population level; that is, declining HIV incidence from an already implemented intervention does not change the delivery cost of another intervention. Rather, our parameters reflect average, aggregate effects in the targeted population. As policymakers move toward a “one stop shop” approach [66], there may be synergies between programs that we do not account for. At the same time, our analysis likely overestimates cost in such a situation by counting the same overhead multiple times. PrEP’s cost, however, comes primarily from the drug itself and is unlikely to decrease if combined with other services. As our analysis suggests that OAT, NSPs, and Test & Treat can be cost-effective even without combining overhead costs or incorporating synergies on an individual level, modeling programs as interdependent would likely not change our general ranking of programs, although it might increase value in an absolute sense if, for instance, adherence to any one program could be improved with multiple enrollment.

Second, certain limitations are inherent to our choice of model. Although extensive sensitivity analysis allows us to investigate parameter sensitivity, the use of a dynamic compartmental model prevents the exploration of structural sensitivities [80,81]. Because compartmental models do not track individuals, we do not explicitly model such phenomena as loss to follow-up in HIV care, although we do calibrate linkage rates to account for long-term drop-offs in the care cascade [27,39,40]. Our model does not explicitly account for networks or distinguish risk on an individual basis. All of the interventions we consider would be more cost-effective if targeted to individuals central to injecting or sexual networks. Because an individual’s decrease in needle sharing must also affect the number of shared needles of his or her injecting partners, the effects of NSPs, in particular, may be underestimated by a compartmental model, where we can only estimate average effectiveness for the individual accessing the service. However, as we find NSPs to be cost-effective under most circumstances, and as we find the most substantial benefit to come from direct, individual health gains accruing from OAT, independent of network effects, this simplification is not likely to alter our model’s general findings.

Third, we assume constant returns to scale (i.e., per person costs do not increase as coverage expands) even though, in practice, enrolling more marginalized PWID, especially at higher coverage levels, is likely more costly. While diminishing returns as coverage rises may decrease the value of investments in absolute terms, the relative ranking of programs, assuming that marginalized PWID are equally difficult to enroll in any program, would not be affected. Moreover, our framing of low, medium, and high coverage levels is meant to be illustrative of possible investment patterns, but our prioritization rankings and cost-effectiveness conclusions do not change with arbitrary definitions of “high” coverage (S1 Appendix, Section 5.2.3). In practice, achievable coverage levels may vary extensively by region. However, our cost-efficient frontier (Fig 2) suggests that OAT investments along a range of feasible coverage levels can provide high value, even when such levels are below 50%.

Finally, many of our model parameters are uncertain. To address this uncertainty, we calibrated our model to empirical data and conducted extensive sensitivity and uncertainty analyses (S1 Appendix, Section 5). Although these analyses suggest that our main findings are consistent across many scenarios and analyses, there remains uncertainty about HIV transmission dynamics and the effectiveness of prevention programs when implemented jointly. Our findings should be interpreted in view of these limitations.

Our model-based analysis builds intuition for the mechanisms behind the conclusions of Des Jarlais et al. [9], who found that a combined prevention approach was effective in ending HIV epidemics among PWID in a number of settings. We also project a range of scenarios under which these combined portfolios can be cost-effective. Where budgets are limited, our analysis suggests that a reasonable approach is for resources to be allocated first towards expanding OAT coverage, which we assume can have additional quality-of-life benefits for PWID beyond HIV prevention, including cessation of drug use. Combining NSP scale-up with OAT expansion, and investing remaining budget resources in further NSP expansion and in Test & Treat, has the potential to be both cost-effective and beneficial to the entire population. While budgets may dictate the extent to which programs can be scaled, we project that the relative ranking among modeled programs remains consistent across varied delivery contexts. Investment in cost-effective programs is critical given the current epidemic of injection drug use in the US [1,8]. Although further empirical studies of combined prevention programs would be very useful, model-based projections can inform the development of high-value HIV prevention portfolios.

## Supporting information

S1 Appendix Technical supplement. (DOCX)

S1 CHEERS Checklist (PDF)

## Abbreviations

ART: antiretroviral therapy
CDC: US Centers for Disease Control and Prevention
ICER: incremental cost-effectiveness ratio
MSM: men who have sex with men
NSP: needle and syringe program
OAT: opioid agonist therapy
PrEP: pre-exposure prophylaxis
PSA: probabilistic sensitivity analysis
PWID: people who inject drugs
QALY: quality-adjusted life year
Test & Treat: HIV testing and treatment
