# Supplementary material for: Estimation of the cost-effectiveness of HIV prevention portfolios for people who inject drugs in the United States: A model-based analysis
Source: PLoS Med. 2017 May 24;14(5):e1002312. doi: 10.1371/journal.pmed.1002312 (PMC5443477; doi:10.1371/journal.pmed.1002312)
Supplement: S1 Appendix — (DOCX) [file pmed.1002312.s001.docx]

**S1 APPENDIX: TECHNICAL SUPPLEMENT**

**ESTIMATION OF THE COST-EFFECTIVENESS OF HIV PREVENTION PORTFOLIOS FOR PEOPLE WHO INJECT DRUGS IN THE US**

**CL Bernard, DK Owens, JD Goldhaber-Fiebert, ML Brandeau**

**1 HIV Epidemic Model**

We extended a previously published dynamic compartmental model of the HIV epidemic in the United States [1] and programmed it in Matlab R2015b (Mathworks). The model includes adults aged 18 to 64, with preferential mixing between people who inject drugs (PWID), men who have sex with men (MSM), and the remaining heterosexual population considered to be predominately low risk. The model further stratifies the population by awareness of infection, enrollment in opioid agonist therapy (OAT), and enrollment in antiretroviral therapy (ART), and tracks the spread of HIV through sexual and injection-based transmission. The model was empirically calibrated to available US data and all results are averaged across the resulting 182 calibrated sets. We used the model to measure HIV infections averted, HIV prevalence, discounted quality-adjusted life years (QALYs), and discounted costs with and without interventions.

In addition to the dynamic model that captures the costs and effects of the intervention(s) over the 20-year delivery timeline, we linked the population remaining in the model in 2036 (and those maturing out of the model while intervention(s) are active) to a set of Markov models to capture lifetime trajectories in the absence of intervention(s). We implemented the Markov models by running our dynamic compartmental model with entry, maturation, transmission, and intervention turned off and with mortality rates adjusted to reflect the appropriate life expectancy of an individual starting at age 41 (the average age in the model) or 65 (the age at which individuals mature from the model), respectively [2]. Individuals exiting the dynamic model due to maturation or the end of the intervention are moved to the equivalent compartment in the Markov model and transition through states (where associated costs and QALYs accrue) until death. We discounted projected costs and QALYs at 3% annually [3,4]. Extensive documentation on model design, implementation, and calibration for the dynamic and Markov models can be found in the supplemental section of a recently published manuscript [1].

The model’s original design allowed us to assess the costs and benefits associated with introducing HIV pre-exposure prophylaxis (PrEP) for PWID at various coverage levels. The extensions allowed us to turn on or expand other interventions in a similar way so that we could analyze various combinations or portfolios of interventions. These interventions, in addition to PrEP, were OAT, needle-syringe exchange programs (NSP), and Test & Treat, which included additional HIV screening and ART services. All interventions were considered at status quo, low (40% of eligible population), medium (45%), and high (60%) coverage levels. Figure 1 provides a schematic of the modified PWID compartments in the model.

S1 Appendix, Section 2 details model calibration. Sections 3 and 4, respectively, describe the implementation of each intervention singly and in combination with others. S1 Appendix, Section 5 presents details of our sensitivity analyses.

**2 Calibration**

The model calibration has been published in the supplemental material of a recent manuscript [1]. We summarize that discussion here. We used beta, gamma, Dirichlet, and (truncated) log-normal distributions to model the uncertainty around 99 epidemiologic inputs. We began with wide confidence intervals to reflect parameter uncertainty as well as population heterogeneity and generated 15 million sets of model parameters with a random search algorithm that simultaneously sampled from those distributions [5,6]. We simulated the model and tracked 115 epidemiologic targets (multiple demographic cross-sections multiple times over a 9-year time horizon) for each parameter set. Because many combinations of parameters resulted in implausible outputs, we used exclusion criteria as well as an objective function that fit our targets simultaneously [5,7,8]. This resulted in 182 best-fitting parameter sets over which all analyses were conducted and then averaged for final results [9].

**3 Single Interventions**

**3.1 OAT**

**3.1.1 OAT Implementation**

OAT is a separate compartment stratification in the model. Individuals leave OAT either by quitting drug use and moving to a lower risk group or, in the majority of cases, quitting OAT itself. Under the status quo, the model maintains the OAT population by specifying the appropriate entry rate to balance the loss of individuals exiting the population. We assumed that, on average across calibrated sets, 24.8% (13.2-34.4%) of PWID were enrolled in OAT in 2015 [10-14]. We considered all PWID to be eligible for OAT, regardless of HIV status.

Interventions that expanded OAT access were implemented by adjusting the entry rate into OAT starting in 2015 so that, within a few years, average OAT coverage across the calibrated sets was at the desired level (40%, 45%, or 50%) and remained so through the year 2035. Because approximately 25% of the eligible population was already receiving OAT, scaling from status quo to low coverage had less relative cost and benefit than scaling from low to medium coverage, for instance. We assumed that OAT interventions affected only the size of the enrolled population and not the cost or effectiveness of OAT for any individual.

**3.1.2 OAT Effectiveness**

In the model, OAT reduced injecting frequency [15,16] and was associated with lower mortality risks [10,17] and higher quality of life (the QALY multiplier, *Q_OAT_*) [10,11,18]. PWID receiving OAT were more likely to be connected to health care and therefore had a higher chance of being screened for HIV and promptly initiating ART if diagnosed [19,20]. OAT thus directly benefited the individual by reducing injection-based risk and time until diagnosis and indirectly benefited the entire population by decreasing the number of injections between sero-discordant partners (especially where the infected partner was not virally suppressed) and therefore the spread of HIV. All effectiveness parameters except for *Q_OAT_* already vary across calibrated sets. Table C in S1 Appendix characterizes the distribution on *Q_OAT_*. One-way sensitivity to *Q_OAT_* is documented in Table A in S1 Appendix and addressed in S1 Appendix, Section 5.1.

**3.1.3 OAT Cost**

We assumed two costs associated with OAT: a $7,000 per person annual cost of enrollment (*C_OAT_*) and a $700 per person up-front, undiscounted start-up cost (*S_OAT_*) necessary to increase capacity of OAT services in the US [10,11,21]. In practice, *S_OAT_* was calculated as an estimated start-up cost per methadone clinic multiplied by the estimated number of new methadone clinics necessary to serve the increased population, which varied to some extent across calibrated sets. We assumed that expanding access to buprenorphine, whose delivery does not require its own clinic, would not require separate start-up costs. Table C in S1 Appendix characterizes the distributions on *C_OAT_* and *S_OAT_*. One-way sensitivities to *C_OAT_* and *S_OAT_* are documented in Table A in S1 Appendix and addressed in S1 Appendix, Section 5.1.

Given the substantial opportunity and transportation-related costs of getting to and from methadone clinics, we would expect *C_OAT_* to decrease as coverage increases [21]. As OAT was already cost-effective in our base case analysis, we did not account for geographic proximity to services, but it should be noted that this simplification biases us against OAT.

**3.2 NSP**

**3.2.1 NSP Implementation**

The model does not include compartmental stratification for NSP. Rather, we assumed that the presence of NSP affected the overall average number of risky injections within the PWID population, as detailed in S1 Appendix, Section 3.2.2. Under the status quo, we assumed negligible presence of NSP nationally [22,23] and calibrated the number of risky injections to current HIV transmission rates to sufficiently reflect the effects of local NSP programs. We considered all PWID to be eligible for NSP, regardless of HIV status.

**3.2.2 NSP Effectiveness**

We assumed that an individual using NSP services would decrease his use of shared injecting equipment by 45% (*E_NSP_*) [24,25]. Like OAT, NSP directly benefits the individual by decreasing the risk of HIV acquisition and indirectly benefits the population by decreasing the number of injections between sero-discordant partners. Unlike OAT, NSP does not lower an individual’s absolute number of injections (only the number of *risky* injections, *R_I_*, defined as the product of total injections, *N_I_*, and percent of injections that use shared equipment, *S_I_*), and therefore does not affect quality of life or mortality risks from overdose.

The following illustrative example of expanding NSP coverage to *x*% of PWID shows how NSP-related interventions affect population-level parameters. The overall number of injections, *N_I_*, would remain unchanged but *x*% of the population would decrease sharing by *E_NSP_*. Thus, if originally *R_I_ = N_I_ S_I_*, the average number of risky injections would become *R_I_ =* (*1-x*)*N_I_ S_I_ + x N_I_ S_I_* (*1- E_NSP_*) under the specified NSP intervention. Thus, the average number of risky injections in the population would decrease by the factor (*1- xE_NSP_*). The percent *x* is a control variable defining the intervention. The term *R_I_*, the product of *N_I_* and *S_I_*, varies across calibrated sets. One-way sensitivity to *E_NSP_* is discussed in S1 Appendix, Section 5.1 and Table A in S1 Appendix. Table C in S1 Appendix characterizes the distribution on *E_NSP_*.

**3.2.3 NSP Cost**

We assumed two costs associated with NSP: a $615 per person annual cost of enrollment (*C_NSP_*) [22,26,27] and the start-up cost (*S_NSP_*) necessary to increase capacity of NSP services in the US. Since the annual cost of enrollment covers the fees of operation for NSP – programs which are usually housed in pharmacies or hospitals or low-infrastructure and/or transportable facilities [28] – we assumed there would not be additional need for facility construction and thus we set *S_NSP_* to zero in the base case. Table C in S1 Appendix characterizes the distributions on *C_NSP_* and *S_NSP_*. One-way sensitivities to *C_NSP_* and *S_NSP_* are documented in Table A in S1 Appendix and addressed in S1 Appendix, Section 5.1.

**3.3 Test & Treat**

**3.3.1 Test & Treat Implementation**

In the model, all individuals who are HIV positive and unaware of their status are subject to background screening rates dependent on risk group and stage of infection [10,29,30]. Individuals initiate treatment and move into a compartment for ART at the time of diagnosis with a probability that is dependent on risk group and stage of infection [30,31]. The Test & Treat program increases HIV detection rates for PWID. Enrollment in ART is a two-step process in the model. First, screening rates are increased. Then, PWID identified under the new screening policy who choose to initiate ART at time of diagnosis (*P_T&T_*, assumed to be 75% in the base case) move into a distinct compartment so that the costs and QALYs specific to the intensive ART program can be calculated (see Figure 1).

We considered all HIV-uninfected and HIV-infected but undiagnosed PWID eligible for screening under a Test & Treat program. Individuals found to be infected were further eligible for treatment. Screening rates were dependent on $P_{T\&T}$; that is, they were calibrated so that, given our assumption on the value of $P_{T\&T}$, average ART enrollment would increase to the desired level at the end of 20 years. Table C in S1 Appendix characterizes the distribution on $P_{T\&T}$. One-way sensitivity to $P_{T\&T}$is discussed in S1 Appendix, Section 5.1 and Table A in S1 Appendix.

Without a Test & Treat intervention, on average 40% of HIV-infected PWID in the model received ART in 2015, with enrollment levels decreasing slightly to 38% by 2035. Thus, in the low coverage scenario, incremental costs and QALYs were slight because the intervention only offset a marginal drop in population. Unlike other interventions, the eligible population for Test & Treat emerges over time, since new individuals cannot be enrolled until they become infected. Therefore, Test & Treat was scaled up in the model over the analytic time horizon (unlike the other interventions, which we assumed could begin substantial scale-up instantaneously).

We discontinued all interventions following an individual’s maturation out of the model or at the end of the 20-year analytic time horizon. We used Markov models to account for additional costs and benefits accruing over the lifetimes of maturing/ remaining individuals in the absence of intervention or transmission. As an example, under the assumption that PrEP would be discontinued for an individual maturing out of the model, anyone uninfected and receiving PrEP in 2035 was assumed to have the same lifetime expected costs and benefits as anyone uninfected and not receiving PrEP in 2035. Details of the Markov models are available in the technical supplement of a previously published paper [1].

The appropriate assignment of lifetime costs and QALYs for individuals receiving ART under a discontinued Test & Treat program was less straightforward and required additional implementation parameters: $P_{T\&T}^{M}$ , the percent of PWID enrolled in the intensive ART arm of the Test & Treat program who remained in ART (but transitioned to standard delivery settings) following maturation from the model (100% in the base case), and $P_{T\&T}^{E}$, the percent remaining at the end of the 20-year analytic time horizon (75% in the base case) [29,30,32,33]. One assumption of a Test & Treat program was that it would identify, recruit, and retain high-risk individuals who would not otherwise be diagnosed, connected to healthcare, or adherent to their treatment. Therefore, costs for the program were higher than the cost of ART alone, and we did not assume that all individuals enrolled would remain on ART in the absence of the intensive patient management of the program. Table C in S1 Appendix characterizes the distributions on $P_{T\&T}^{M}$ and $P_{T\&T}^{E}$. One-way sensitivities to $P_{T\&T}^{M}$ and $P_{T\&T}^{E}$ are documented in Table A in S1 Appendix and addressed in S1 Appendix, Section 5.1.

**3.3.2 Test & Treat Effectiveness**

A Test & Treat program has multiple, substantial benefits to individuals receiving its services and to their injecting and sexual partners. While the diagnosed individual experiences a slight decrease in quality of life in the early stages of HIV [34], we associate awareness of HIV status with a 23.2% decrease in risky injections [10,14] and a 1.66 times increase in condom use [10,12,14,35], both of which benefit the individual’s partners. The greatest benefit of diagnosis is the possibility of getting on treatment. ART is associated with a 16.5 year increase in life expectancy [36-38] and reduces sexual transmission risk by 90% [34,36,39] and injection-based risk by 59% [34,40]. On top of the 15% increase in quality of life that all ART patients receive [18,34,41,42] we included an additional quality adjustment, Q*_T&T_*, set to 3% in the base case, as we assumed that individuals received intensive case management as part of a broader, community-based care initiative [43]. The underlying effectiveness parameters already vary across calibrated sets. Table C in S1 Appendix characterizes the distribution on Q*_T&T_*. One-way sensitivity to Q*_T&T_* is documented in Table A in S1 Appendix and addressed in S1 Appendix, Section 5.1.

**3.3.3 Test & Treat Cost**

The Test & Treat program screens all individuals who do not already have an HIV diagnosis, regardless of whether they are HIV positive or not. Therefore, there are two possible one-time screening and counseling costs: $50 if the patient is uninfected and $500 if the patient is infected [42]. The cost of receiving treatment through a Test & Treat program comes from the $15,000 annual cost of ART [34,41,44,45], C*_ART_*, and the additional $6,600 cost of community-based care and case management [43], C*_T&T_*. We assumed that C*_T&T_* absorbed start-up costs and thus we did not explicitly include an additional cost parameter for Test & Treat. Table C in S1 Appendix characterizes the distributions on C*_ART_* and C*_T&T_*. One-way sensitivities to C*_ART_* and C*_T&T_* are documented in Table A in S1 Appendix and addressed in S1 Appendix, Section 5.1.

**3.4 PrEP**

**3.4.1 PrEP Implementation**

We assumed that no PWID were previously receiving PrEP in 2015. All uninfected PWID were considered eligible to enroll in PrEP and those who became infected while on PrEP remained on PrEP until time of diagnosis. At every time step, after individuals left a PrEP program due to HIV infection, death, or maturation, new individuals were screened and recruited so that a fixed percentage of the population (40%, 45%, or 50% depending on the coverage scenario) remained in the PrEP-specific compartments (see Figure 1). Further details of the implementation of the PrEP intervention are available in the technical supplement of a previously published paper [1].

**3.4.2 PrEP Effectiveness**

PrEP provides direct benefit to the individual by reducing risk of injection- and sexual-based HIV acquisition by *E_PrEP_*, which is 49% in the base case [40]. A previous analysis found that PrEP is most cost-effective when combined with the CDC’s recommendation of frequent screening and toxicity monitoring [46] and with immediate enrollment in ART following diagnosis for those who do become infected [1]. We included both of these policies in our implementation of a PrEP program and, therefore, PrEP had the additional benefit of shortening time from infection to diagnosis to treatment, thereby improving quality of life for the individual and reducing transmission within the population. Table C in S1 Appendix characterizes the distribution on *E_PrEP_*.

**3.4.3 PrEP Cost**

The annual cost of a PrEP program included $800 for screening services, $C_{PrEP}^{S}$, [42,45] and an additional $10,000 for the medication [42,47-49], $C_{PrEP}$. We assumed that a PrEP program would have no start-up costs as services would be delivered in pre-existing clinical settings. Table C in S1 Appendix characterizes the distributions on $C_{PrEP}^{S}$ and $C_{PrEP}$. One-way sensitivity to $C_{PrEP}$is documented in Table A in S1 Appendix and addressed in S1 Appendix, Section 5.1.

**4 Intervention Portfolios**

Interventions implemented in combination were assumed to be independent; that is, we did not adjust costs or effects of the individual programs nor did we explicitly track the number of individuals receiving services from multiple programs whose infections could not be prevented twice (leading to a possible overestimate of synergistic benefit). In the model, this meant that every portfolio was implemented simply by turning on or off each component intervention. We associated this with the real-world scenario in which policy makers would invest in separate programs which would continue to operate, whether in tandem or alone, with the same budgeting and recruitment strategies. In practice this meant that often the combined effects of programs resulted in coverage scenarios higher than the targeted 40%, 45%, or 50% (leading to a possible overestimate of net cost). For instance, low coverage of Test & Treat combined with low coverage of OAT resulted in ART coverage above 40% because individuals on OAT are more likely to get on ART and enrollment rates for Test & Treat were not decreased to reflect this. The possible biases towards effectiveness and cost raised here are addressed in the manuscript’s Discussion section.

**5 Sensitivity Analysis**

As discussed in S1 Appendix, Section 2, most underlying parameters in the model vary across calibrated sets. The sensitivity of our results to these parameters is discussed in S1 Appendix, Section 5.3. Parameters dealing explicitly with the uncalibrated cost and effectiveness parameters of interventions are explored in S1 Appendix, Section 5.1. We address sensitivities to the definition and implementation of interventions in S1 Appendix, Section 5.2. S1 Appendix, Section 5.4 discusses the results of probabilistic sensitivity analysis.

**5.1 Intervention Parameters**

Table A in S1 Appendix summarizes the results of multiple one-way sensitivity analyses to our key intervention parameters. Ranges for these parameters were not necessarily chosen to reflect 95% confidence intervals but rather to illustrate the degree of sensitivity to the parameter or to demonstrate lower- or upper-bounding scenarios. The analyses were generated to develop intuition and thus should be taken as instructive rather than prescriptive.

While the relative ordering of interventions does change under certain circumstances and incremental cost-effectiveness ratios (ICERs) show slight to moderate variation, the results largely confirm the base-case analysis. Even with high start-up costs for NSP implementation, which we assumed to be minimal in our base-case analysis, NSP remains cost-effective, although at very high per-person cost estimates or very low efficacy estimates, increasing NSP coverage can exceed $75,000 per QALY gained. Within feasible ranges of OAT delivery and start-up costs, OAT is still cost-effective, costing from $13,000 to $25,000 per QALY gained. In the latter case, the highest-value portfolio would first add NSP to high coverage levels and then move directly to adding high coverage of OAT. In the base case analysis, NSP coverage is increased to 50% before Test & Treat is added to the portfolio. A few one-way sensitivity analyses (e.g., high start-up cost for NSP) described above reverse this relative ranking by making Test & Treat scale-up a higher priority than NSP.

These sensitivity analyses underscore our projections that OAT is a valuable investment while PrEP, even under favorable cost estimates, is not likely to be. Under many delivery scenarios, we estimate that NSP can surpass OAT as the priority investment and, as NSP requires the lowest budget outlays of any prevention program, it is sufficiently cost-effective to merit concurrent expansion under most circumstances. Test & Treat is a generally cost-effective supplement to any prevention portfolio but, unlike OAT and NSP, our model does not project it to be the first intervention to scale up. Further discussion of our findings and their practical implications can be found in the manuscript’s Sensitivity Analysis section.

**5.2 Intervention Implementation**

It is difficult to compare interventions without a model, and even within a model, biases may emerge depending on how we define and implement each intervention. Below we discuss various definitions of coverage and approaches to portfolio construction, demonstrating the consistency of results across standardized coverage and investment levels as well as alternative analytic time horizons.

**5.2.1 Standardization of Enrolled Population**

As both the size of the eligible population and the status quo coverage level in 2015 vary across each of the four interventions considered, one potential concern with our base case implementation is that we are “comparing apples and oranges” when it comes to affected populations. It therefore makes sense to standardize the size of the population directly affected by each intervention at each coverage level. If we define low coverage as reaching *X* people, then, for example, a low-coverage PrEP intervention will result in *X* PWID on PrEP. A low-coverage OAT intervention will result in *X + X_0_ ^OAT^* receiving OAT, where *X_0_ ^OAT^* is the status quo OAT population.

A drawback of standardizing the affected population in this way is that a Test & Treat program becomes the limiting intervention because its eligible population is by far the smallest, assuming that, on average 10% of PWID are HIV positive [12]. Therefore, we were constrained to defining coverage in increments of 30,000 people (which is less than 3% of the estimated US PWID population size) [50-52]: low coverage is 30,000 PWID reached; medium, 60,000; high, 90,000. Even at these relatively low numbers, the model could not meet high targets for Test & Treat, and, in practice, the number of individuals reached was closer to 70,000. At the same time, PrEP coverage was so low that its non-linear benefits to the larger PWID population, such as approximating herd immunity, were restricted. Thus, while standardizing the size of the population reached by each intervention is in theory a good approach for reducing potential bias, implementation difficulties led us to not choose this approach in the main analyses.

It is reassuring to note that when we performed analyses with this alternate definition we reached the same conclusions as in our main analyses. Table B in S1 Appendix gives the cost-efficient frontier when interventions are standardized by affected population. A highest-value portfolio begins with maximizing OAT coverage followed by maximizing NSP coverage, a result with implications similar to those from many of our one-way sensitivity analyses, which suggest that concurrent investment in OAT and NSP is favorable under most circumstances. We would then recommended investment in Test & Treat, with all combinations of the three interventions considered to be highly cost-effective with ICERs less than $50,000 per QALY gained. We would not recommend scaling up PrEP coverage as its ICER is close to $500,000 per QALY gained.

**5.2.2 Standardization of Budget**

Another way to try to minimize bias is to standardize the budget impact for each coverage level across interventions. We considered a stylized example where we assumed that a policy maker could make investments at four possible coverage levels or caps: *$X*, *$2X*, *$3X*, or *$4X*. Each intervention could be invested in at increments of *$X*. Thus at a cap of *$X*, we could only choose to invest in one of the interventions at a time and had four choices. At a *$2X* cap we could invest *$2X* in one intervention or *$X* into two interventions. At a cap of *$nX* we had $\Sigma{}_{i=1}^{n}\left( \begin{matrix} 4 \\ n \end{matrix} \right)$ possible portfolios to choose from.

While this standardization also had its advantages, we ran into a similar issue of having one limiting intervention, which in this case was NSP. Because NSP is far less expensive than the other interventions, we were constrained by feasible levels of investment one could make in such a program. Thus, we chose *$X* to be $750 million over 20 years, and *$4X* is only $3 billion, which is substantially lower than any 20-year investment a federal policy maker would be considering. Still, the results serve to reinforce our understanding of the relative ranking of interventions.

Figure B in S1 Appendix plots the incremental QALYs for all of the possible portfolio choices at each budget impact level. OAT consistently yields the highest number of QALYs and PrEP the lowest, with NSP and Test & Treat performing at comparable levels. It is important to note the distinction between budget impact and incremental costs. We define budget impact to be the upfront cost directly spent on establishing and maintaining a given intervention. However, in net, society may end up spending more or less as downstream costs are offset or incurred as a result of the program. In the case of OAT, incremental costs are higher than budget impact because OAT indirectly increases ART enrollment. For NSP, incremental costs are less than budget impact due to the cost savings from potential HIV infections averted. Thus, while Figure B in S1 Appendix is useful for intuition, it does not necessarily mean that ICERs will rank in the same way. Further analysis does confirm, however, that they do. Considered singly at the $750 million investment cap, OAT is the highest-value intervention at $18,000 per QALY gained. NSP and ART have almost identical ICERs just under $30,000 per QALY gained, and PrEP has an ICER of around $275,000.

Figure C in S1 Appendix plots HIV infections averted for all of the possible portfolio choices at each budget impact level. NSP outperforms the other interventions, and OAT ranks last. It is important to remember that this standardization approach effectively maximizes NSP coverage, while the other interventions are quite limited in scale. The figure provides further motivation for investing in NSP while maximizing OAT coverage since NSP is consistently the least expensive intervention and can complement the services that OAT provides.

**5.2.3 Standardization of Percent Eligible Population**

For our base analysis we standardized across the percent of the eligible population covered for each program. An initial version of the analysis (Figure A in S1 Appendix) considered low, medium, and high coverage at 40%, 60%, and 80%, respectively. Although 80% is optimistically high for PWID coverage, we considered this level in order to demonstrate an upper bounding scenario. Capping enrollments at 60% or 40% levels did not alter the relative ranking of programs. Our base case analysis uses more realistic enrollment levels (40%, 45%, and 50% of the eligible population), but findings from our base case and sensitivity analyses show consistency across the levels chosen for low, medium, and high coverage.

**5.2.4 Duration of Intervention (Analytic Time Horizon)**

It is possible that each intervention works on a different time scale and that the 20-year analytic time horizon we chose may bias us towards one intervention or another. (Note, here the analytic time horizon refers exclusively to the time over which the intervention is active. All analyses except the one described in the paragraph below included lifetime costs and benefits.) We performed our analysis over a number of time horizons ranging from 5 to 50 years. We found no change in intervention ranking over shorter time horizons. That is, even if the direct benefits of a program are short lived, the programs still rank in the same order and with similar ICERs. Over longer time horizons (which, in comparison to the base case analysis, depend less on lifetime costs/benefits from the Markov models because of discounting and essentially provide longer-lasting benefits to individuals), the highest-value portfolio would first expand NSP to medium coverage levels and then begin expanding OAT. Test & Treat is moderately cost-effective, and PrEP was not likely to be cost-effective. These findings reinforce the robustness of the conclusions in the manuscript.

Furthermore, we examined a scenario that only tracks benefits over the analytic time horizon of 20 years in the model and excludes lifetime trajectories (we do not add in the costs and benefits resulting from the additional Markov model) and found that OAT was still the priority investment, with additions of NSP and PrEP looking less favorable than in the base case analysis. This indicates that the primary advantages of NSP and PrEP, that is, infections averted, accumulate over a lifetime but have less immediate effect than OAT and Test & Treat, which provide direct QALY gains to enrolled individuals.

**5.3 Underlying Model Parameters**

Because all analyses were performed across 182 calibrated sets, for each parameter we were able to determine the highest-value portfolio when we sampled only from the sets with parameter values in the lowest or highest 5% among all sets. We did not observe substantial variation in results beyond the slight alterations in relative rankings and/or ICERs described in S1 Appendix, Section 5.1.

Table 1 of the main manuscript enumerates key underlying parameters and their ranges among calibrated sets. Below we address the underlying motivation for and implications of notably wide ranges on several parameters.

*Percent decrease in risky injections from OAT*

We used the wide confidence interval of 0.004-82.2% for the percent decrease in risky injections due to OAT to account for the underlying importance of correct dosing. Evidence in the US shows that, currently, 56% of all patients, served by one-third of all facilities, receive insufficient dosing, and expanding OAT services, especially in areas with historically conservative views around abstinence, might only increase this proportion [53].

We were concerned that studies on OAT effectiveness might have controlled delivery settings not reflective of wider dosing practices. Thus, to be cautious about biasing towards this intervention, we included the possibility that OAT could be almost completely ineffective in terms of reducing injections, while still having other benefits such as linking individuals to further care.

A meta-analysis on the effects of opioid substitution therapy estimated a 54% (0.32, 0.67) reduction in risk of acquiring HIV [15]. Since we wanted to reflect the point estimate across calibrated sets and were using a truncated log-normal distribution to characterize uncertainty for this hazard ratio, including the very low-end estimate pushed the high-end estimate above the range in [15], but using a wider range in our full analysis did not affect our conclusions. (Performing the analysis on the calibrated 97 sets whose parameter on OAT effectiveness falls into this narrower range gave identical program rankings and almost identical ICER results to our full analysis.) Specifically looking at calibrated sets with parameter value falling in the bottom 5% of the effectiveness range also did not alter our conclusions.

To further examine sensitivity to this parameter, we modified all sets and artificially set their OAT-related reductions in injecting frequency to 0. Conducting our analysis on these sets we projected that NSP would be the priority investment but that OAT (at $19,000 per QALY gained) and Test & Treat (at $31,000 per QALY gained) were both cost-effective additions to the portfolio, while PrEP (at $417,000 per QALY gained) was not.

*Decrease in risky injections due to awareness of HIV status*

There is limited data on extent to which awareness of HIV status decreases risky injections. This uncertainty is reflected in our range of 0-55%. A previous modeling study reported a confidence interval of 5-30% [10], while an epidemiologic study found no change in sharing and high-risk sex between infected (aware or not) and uninfected PWID/MSM [14]. This highest-risk group might not be representative of all PWID. Nonetheless we wanted to include this lower-end estimate while still allowing for an approximately 20% point estimate of risk reduction, consistent with [10]. We used a truncated log-normal distribution to characterize uncertainty for this hazard ratio, and this pushed the high-end estimate above the range in [10].

The use of calibrated sets with parameters falling in the top 5% or bottom 5% of this range did not lead to any substantial change in conclusions. Program rankings remained the same although, at the high end of risk reduction, the ICER for Test & Treat dropped by $9,000 (in either case, it would be considered cost-effective at a $50,000 willingness-to-pay threshold) and subsequent NSP scale-up looked less desirable.

To further examine sensitivity to this parameter, we modified all sets and artificially set their awareness-related reductions in injection equipment sharing to 0. Conducting our analysis on these sets we projected that NSP would be the priority investment but that OAT (at $18,000 per QALY gained) and Test & Treat (at $35,000 per QALY gained) were both cost-effective additions to the portfolio, while PrEP (at $505,000 per QALY gained) was not.

*Joint distribution*

No published data are available to characterize a joint distribution for the reduction in risky injections due to OAT and the reduction due to awareness of HIV status. The OAT parameter speaks to an aspect of program implementation whereas the decrease in risky injections, while associated with a Test & Treat program that increases awareness, speaks to behavior particular to an individual (independent of enrollment in a Test & Treat program), so there is nothing to suggest that these parameters should have a strong correlation. Over all 182 calibrated sets, these parameters have a correlation of -0.09.

It is nonetheless reasonable to consider what would happen in the case that both parameters are at the lower end of their ranges. There are 3 calibrated sets for which OAT effectiveness is less than 32% and the decrease in risky injections due to awareness is less than 5%. We performed the analysis on these sets and found the following recommendations: begin with NSP scale-up at approximately $13,000/QALY gained, then scale up OAT at approximately $19,000/QALY gained, then scale up Test & Treat at approximately $44,000/QALY gained. Additions of PrEP cost over $450,000/QALY gained. This result yields three main takeaways consistent with other sensitivity analyses: 1) NSP can replace OAT as the priority investment under circumstances unfavorable to OAT. 2) While ICERs can vary, the top three programs (NSP, OAT, and Test & Treat) are cost-effective under a $50,000 willingness-to-pay threshold. 3) Even under circumstances unfavorable to OAT and Test & Treat, PrEP is not likely to be a cost-effective investment.

We conducted PSA on these 3 calibrated sets, sampling intervention implementation parameters (e.g., costs, QALYs) over each set 30 times (Table D2 in S1 Appendix) [54]. Over these 90 model instantiations, we projected that OAT was the priority investment 63% of the time and NSP was the priority investment 37% of the time. The percentage of times when OAT was a cost-effective addition to the portfolio remained at 100%, but times when Test & Treat and NSP were a cost-effective addition increased a few percentage points at every willingness-to-pay threshold, and times when PrEP was a cost-effective addition increased from 1% to 2.2% at a willingness-to-pay threshold of $150,000.

To further examine joint sensitivity to these parameters, we modified all sets and artificially set both their OAT-related reductions in injecting frequency and their subsequent reductions in injection equipment sharing following a positive HIV diagnosis to 0. Conducting our analysis on these sets, we projected that NSP would be the priority investment but that OAT (at $19,000 per QALY gained) and Test & Treat (at $32,000 per QALY gained) were both cost-effective additions to the portfolio, while PrEP (at $336,000 per QALY gained) was not.

We conducted PSA on all modified sets, sampling intervention implementation parameters (e.g., costs, QALYs) over each 30 times (Table D3 in S1 Appendix) [54]. With over 5,000 samples, we projected that OAT was the priority investment 43% of the time and NSP was the priority investment 57% of the time. The percentage of times when OAT was a cost-effective addition to the portfolio remained at 100%, but the percentage of times when NSP was a cost-effective addition increased from 74% to 94% at the willingness-to-pay threshold of $50,000, times when Test & Treat was a cost-effective addition increased a few percentage points at every willingness-to-pay threshold, and times when PrEP was a cost-effective addition increased from 1% to 14% at a willingness-to-pay threshold of $150,000.

We conclude that our model projections are not highly sensitive to either parameter, but that there could be circumstances where both OAT and Test & Treat look less favorable and when PrEP’s value might increase. These findings help to develop the intuition for why we estimate that OAT can be the highest-value investment: the competing mortality rates are so high for PWID that interventions that immediately increase quality of life provide the most value. The model is less sensitive to intervention parameters that relate to HIV transmission because reducing HIV incidence is not the driving cause of health benefit in the model.

**5.4 Probabilistic Sensitivity Analysis**

Table C in S1 Appendix shows the beta, gamma, and (truncated) log-normal distributions used to characterize the uncertainty around intervention parameters. (Distributions on calibrated model parameters can be found in the supplement of a previously published paper [1].) For each of the 182 calibrated sets, we simultaneously sampled all uncalibrated model parameters from their respective distributions 30 different times, resulting in a probabilistic sensitivity analysis with more than 5,000 draws. We excluded sampled sets that resulted in infeasible real-world scenarios.

Our results are not highly sensitive to parameter or second-order uncertainty (Table D1 in S1 Appendix) [54]. There were no sampled sets for which the model selected Test & Treat or PrEP to be first additions to a portfolio and only 1% of all sampled sets had an optimal portfolio to which PrEP would ever be a cost-effective addition at a $150,000 willingness-to-pay threshold. 41% of sampled sets added NSP to the portfolio before adding OAT. Additions of OAT, NSP, Test & Treat, and PrEP to the optimal portfolio cost less than $50,000 per QALY gained in 100%, 74%, 4.7%, and 0.5% of all PSA samples, respectively. When the threshold was $100,000 per QALY gained, this changed to 100%, 93%, 33%, and 0.5% of all PSA samples, respectively. When the threshold was $100,000 per QALY gained, this changed to 100%, 97%, 67%, and 1.0% of all PSA samples, respectively. These results are consistent with our general projections that OAT and NSP can be cost-effective (we recommend that they be scaled simultaneously), with the addition of Test & Treat generally considered cost-effective and the addition of PrEP unlikely to be considered cost-effective.

**6 Limitations and Future Work**

A discussion of the most pertinent limitations of our analysis can be found in the main text. An additional limitation is that we did not include hepatitis C virus (HCV) in the model, which likely biases us against both OAT and NSP as these interventions decrease injecting and sharing risks. We found both interventions to be highly cost-effective without explicitly accounting for HCV, and the high mortality rate for PWID in the model implicitly incorporates competing mortality risks that might otherwise cause us to overestimate the benefit from preventing an HIV infection. One area of future work is to incorporate HCV and HIV into a comorbidity model that explicitly captures competing mortality risks and intervention tradeoffs. Table E in S1 Appendix further enumerates the assumptions and limitations of our analysis.

**Table A: Sensitivity to Intervention Parameters**

| **Parameter** | **Base case** | **Low value** | **Portfolio****^[[1]](#footnote-1)^** | **OAT ICER****^[[2]](#footnote-2)^** | **NSP ICER**† | **T&T ICER**† | **PrEP ICER**† |
| --- | --- | --- | --- | --- | --- | --- | --- |
| *Q_OAT_* | 1.06 | 1.00 | [1000], [2000], [3000], [3100], [3200], [3300], [3330], [3331], [3332], [3333] | $17,000 | $27,000 | $36,000 | $666,000 |
| *C_OAT_* | $7,000 | $4,320 | [3000], [3100], [3200], [3300], [3310], [3330], [3331], [3332], [3333] | $13,000 | $27,000 | $35,000 | $612,000 |
| *S_OAT_* | $700 | $350 | [1000], [2000], [3000], [3100], [3200], [3300], [3310], [3330], [3331], [3332], [3333] | $18,000 | $28,000 | $34,000 | $613,000 |
| *E_NSP_* | 45% | 20% | [1000], [2000], [3000], [3030], [3130], [3230], [3330], [3331], [3332], [3333] | $18,000 | $79,000 | $32,000 | $518,000 |
| *C_NSP_* | $615 | $308 | [0100], [0200], [0300], [3300], [3310], [3330], [3331], [3332], [3333] | $19,000 | $5,000 | $34,000 | $613,000 |
| *S_NSP_* | $0 | $0 | [1000], [2000], [3000], [3030], [3330], [3331], [3332], [3333] | $18,000 | $36,000 | $32,000 | $613,000 |
| *P_T&T_* | 75% | 50% | [1000], [2000], [3000], [3100], [3200], [3300], [3310], [3330], [3331], [3332], [3333] | $18,000 | $28,000 | $36,000 | $588,000 |
| *P^M^_T&T_* | 100% | 30% | [1000], [2000], [3000], [3100], [3200], [3300], [3310], [3330], [3331], [3332], [3333] | $18,000 | $28,000 | $34,000 | $613,000 |
| *P^E^_T&T_* | 75% | 30% | [1000], [2000], [3000], [3100], [3200], [3300], [3310], [3320], [3330], [3331], [3332], [3333] | $18,000 | $28,000 | $32,000 | $598,000 |
| Q*_T&T_* | 3% | 0% | [1000], [2000], [3000], [3100], [3200], [3300], [3310], [3330], [3331], [3332], [3333] | $18,000 | $28,000 | $35,000 | $608,000 |
| C*_ART_* | $15,000 | $9,170 | [1000], [2000], [3000], [3030], [3130], [3230], [3330], [3331], [3332], [3333] | $18,000 | $31,000 | $25,000 | $615,000 |
| C*_T&T_* | $6,600 | $3,300 | [1000], [2000], [3000], [3030], [3130], [3230], [3330], [3331], [3332], [3333] | $18,000 | $30,000 | $28,000 | $614,000 |
| C*_PrEP_* | $10,000 | $1,000 | [1000], [2000], [3000], [3100], [3200], [3300], [3310], [3320], [3330], [3331], [3332], [3333] | $18,000 | $28,000 | $34,000 | $92,000 |
|  |  |  |  |  |  |  |  |
| **Parameter** | **Base case** | **High value** | **Portfolio*** | **OAT ICER**† | **NSP ICER**† | **T&T ICER**† | **PrEP ICER**† |
| *Q_OAT_* | 1.06 | 1.06 | [1000], [2000], [3000], [3100], [3200], [3300], [3310], [3320], [3330], [3331], [3332], [3333] | $18,000 | $28,000 | $34,000 | $613,000 |
| *C_OAT_* | $7,000 | $10,430 | [0100], [0200], [0300], [3300], [3330], [3331], [3332], [3333] | $25,000 | $20,000 | $37,000 | $613,000 |
| *S_OAT_* | $700 | $1,400 | [1000], [2000], [3000], [3100], [3200], [3300], [3310], [3330], [3331], [3332], [3333] | $18,000 | $28,000 | $34,000 | $613,000 |
| *E_NSP_* | 45% | 80% | [0100], [0200], [0300], [3300], [3310], [3330], [3331], [3333] | $19,000 | $8,000 | $35,000 | $785,000 |
| *C_NSP_* | $615 | $1,230 | [1000], [2000], [3000], [3030], [3130], [3230], [3330], [3331], [3332], [3333] | $18,000 | $71,000 | $32,000 | $613,000 |
| *S_NSP_* | $0 | $3,600 | [1000], [2000], [3000], [3030], [3330], [3331], [3332], [3333] | $18,000 | $43,000 | $32,000 | $613,000 |
| *P_T&T_* | 75% | 90% | [1000], [2000], [3000], [3100], [3200], [3300], [3330], [3331], [3332], [3333] | $18,000 | $28,000 | $33,000 | $625,000 |
| *P^M^_T&T_* | 100% | 100% | [1000], [2000], [3000], [3100], [3200], [3300], [3310], [3320], [3330], [3331], [3332], [3333] | $18,000 | $28,000 | $34,000 | $613,000 |
| *P^E^_T&T_* | 75% | 100% | [1000], [2000], [3000], [3100], [3200], [3300], [3310], [3330], [3331], [3332], [3333] | $18,000 | $28,000 | $35,000 | $622,000 |
| Q*_T&T_* | 3% | 7% | [1000], [2000], [3000], [3100], [3200], [3300], [3310], [3330], [3331], [3332], [3333] | $18,000 | $28,000 | $33,000 | $618,000 |
| C*_ART_* | $15,000 | $22,300 | [1000], [2000], [3000], [3100], [3200], [3300], [3310], [3320], [3330], [3331], [3332], [3333] | $18,000 | $27,000 | $43,000 | $610,000 |
| C*_T&T_* | $6,600 | $13,200 | [1000], [2000], [3000], [3100], [3200], [3300], [3310], [3330], [3331], [3332], [3333] | $18,000 | $28,000 | $42,000 | $612,000 |
| C*_PrEP_* | $10,000 | $10,000 | [1000], [2000], [3000], [3100], [3200], [3300], [3310], [3320], [3330], [3331], [3332], [3333] | $18,000 | $28,000 | $34,000 | $613,000 |

**Table B: Cost-Efficient Frontier, Interventions Standardized by Affected Population**

| **Intervention Portfolio^[[3]](#footnote-3)^** | **Total Costs**  ***($, Billion)*** | **Total QALYs**  **(Billion)** | **Incremental Costs**  ***($, Billion)*** | **Incremental QALYs (Thousands)** | **ICER** |
| --- | --- | --- | --- | --- | --- |
| Status Quo | 32,528 | 6.4340 | -- | -- | -- |
| Low OAT | 32,532 | 6.4342 | 3.93 | 218.5 | $18,000 |
| Medium OAT | 32,536 | 6.4344 | 4.01 | 223.5 | $18,000 |
| High OAT | 32,540 | 6.4347 | 4.47 | 249.0 | $18,000 |
| High OAT, Low NSP | 32,541 | 6.4347 | 0.22 | 6.8 | $32,000 |
| High OAT, Medium NSP | 32,541 | 6.4347 | 0.22 | 6.8 | $32,000 |
| High OAT, High NSP | 32,541 | 6.4347 | 0.22 | 6.7 | $32,000 |
| High OAT, High ART | 32,573 | 6.4356 | 31.97 | 925.4 | $35,000 |
| High OAT, High ART, Low NSP | 32,573 | 6.4356 | 0.20 | 4.7 | $43,000 |
| High OAT, High ART, Medium NSP | 32,573 | 6.4356 | 0.20 | 4.6 | $43,000 |
| High OAT, High ART, High NSP | 32,574 | 6.4357 | 0.20 | 4.5 | $43,000 |
| High OAT, High ART, High NSP, High PrEP | 32,588 | 6.4357 | 14.45 | 30.9 | $468,000 |

**Table C: Distributions on Additional^[[4]](#footnote-4)^ Intervention Parameters**

|  | **Distribution^[[5]](#footnote-5)^** | |
| --- | --- | --- |
| **Model Parameter** | **Type** | **Parameters** |
| **Opioid Agonist Therapy** | |  |
| *C_OAT_* | Gamma | a=20; b=29.2 |
| *S_OAT_* multiplier | log-normal | μ =-0.26; σ=0.72 |
| *Q_OAT_* | Truncated log-normal | μ=-0.054; σ=0.074 |
| **Needle-Syringe Exchange Program** |  |  |
| *E_NSP_* | Truncated log-normal | μ =-0.86; σ=0.31 |
| *C_NSP_* | Gamma | a=3; b=2.1 |
| *S_NSP_* | Gamma | a=5; b=1.6 |
| **Test & Treat** |  |  |
| *P_T&T_* | Beta | a=10; b=3.3 |
| $P_{T\&T}^{M}$ | Beta | a=2; b=0.5 |
| $P_{T\&T}^{E}$ | Beta | a=3; b=1.0 |
| *Q_T&T_* | Truncated log-normal | μ=0.03; σ=0.03 |
| *C_ART_* | Gamma | a=20; b=62.5 |
| *C_T&T_* | Gamma | a=5; b=13.2 |
| **PrEP** | |  |
| *E_PrEP_* | Truncated log-normal | μ=-0.90; σ=0.67 |
| $C_{PrEP}^{S}$ | Gamma | a=2; b=33.3 |
| *C_PrEP_* | Gamma | a=40; b=20.8 |

**Table D1: Statistics from Probabilistic Sensitivity Analysis**

|  | **Priority Investment****^[[6]](#footnote-6)^** | **$50,000****^[[7]](#footnote-7)^** | **$100,000**† | **$150,000**† |
| --- | --- | --- | --- | --- |
| **OAT** | 59% | 100% | 100% | 100% |
| **NSP** | 41% | 74% | 93% | 97% |
| **Test & Treat** | 0% | 4.7% | 33% | 67% |
| **PrEP** | 0% | 0.5% | 0.5% | 1.0% |

**Table D2: Statistics from Probabilistic Sensitivity Analysis, Selected Calibrated Sets^[[8]](#footnote-8)^**

|  | **Priority Investment*** | **$50,000**† | **$100,000**† | **$150,000**† |
| --- | --- | --- | --- | --- |
| **OAT** | 63% | 100% | 100% | 100% |
| **NSP** | 37% | 76% | 96% | 99% |
| **Test & Treat** | 0% | 7.8% | 40% | 69% |
| **PrEP** | 0% | 0% | 0% | 2.2% |

**Table D3: Statistics from Probabilistic Sensitivity Analysis, Altered Calibrated Sets^[[9]](#footnote-9)^**

|  | **Priority Investment*** | **$50,000**† | **$100,000**† | **$150,000**† |
| --- | --- | --- | --- | --- |
| **OAT** | 43% | 100% | 100% | 100% |
| **NSP** | 57% | 94% | 99% | 100% |
| **Test & Treat** | 0% | 7.9% | 37% | 68% |
| **PrEP** | 0% | 0.5% | 3.2% | 14% |

**Table E: Key Assumptions and Limitations in Intervention Implementation**

| **Assumption/Limitation** | **Explanation** |
| --- | --- |
| OAT is the only mechanism through which PWID can move to lower-risk groups. | [11,55] |
| OAT and (awareness and) ART provide direct health benefits to the individual. | [10,11,17-20] and [34,40] [10,12,14,30,34-39] |
| NSP has negligible scale-up costs in the base case. | Because NSPs can be integrated into already existing locations (pharmacies, hospitals) or are mobile in the community, we assumed that they did not require the construction of new facilities or face the same zoning restrictions as OAT clinics. We therefore assumed that starting costs would be nominal and absorbed into the operational budgets for NSPs in the first year [22,26,27]. We explore this assumption in sensitivity analysis. |
| ART levels rise gradually over the time horizon whereas OAT, NSP, and PrEP programs scale rapidly. | Because an infection must occur before enrollment in Test & Treat, this intervention, unlike others, cannot scale immediately in the model. |
| PrEP is delivered with the CDC’s clinical guidelines (e.g., HIV screening every 3 months, toxicity monitoring every 6 months) and with prompt and sustained provision of ART for those who do become infected. | PrEP has highest value under this delivery scenario [46]. An assumption of different PrEP delivery would make PrEP appear less favorable than in our analyses. |
| Combinations of programs are implemented as independent. | This assumption might cause us to underestimate effectiveness because of program synergies for the individual enrolled in multiple programs or overestimate overhead cost. However, PrEP’s high drug cost is unlikely to come down if delivered in the context of other programs. Adherence to PrEP might improve with multiple enrollment, but even with improved adherence and high estimates of PrEP efficacy, PrEP is not likely to be cost-effective [46]. Thus, modeling independent programs does not unduly bias against PrEP in the program ranking, although PrEP might look more favorable in an absolute sense. Our model suggests that OAT, NSP, and Test & Treat can be cost-effective even without combining overhead costs or incorporating synergies on an individual level, so modeling programs as interdependent would likely not change our general ranking of programs. |
| Use of dynamic compartmental model prohibits tracking individuals. | We do not explicitly model individual-level phenomena such as loss to follow up in HIV care, although we do calibrate linkage rates to account for long-term drop-offs in the care cascade [10,29,30]. |
| The compartmental model does not account for networks. | All of the interventions we consider would be more cost-effective if targeted to individuals central to injecting or sexual networks. Because an individual’s decrease in needle sharing must also affect the number of shared needles of his injecting partners, the effects of NSP, in particular, may be underestimated by a compartmental model where we can only estimate average effectiveness for the individual accessing the service. However, as we find NSP to be cost-effective under most circumstances, and as we find the most substantial benefit of OAT to come from direct, individual health gain, independent of network effects, this simplification may have less effect on our model’s general findings than in other transmission contexts. |
| Cannot explore structural sensitivities with our dynamic compartmental model. | Future work is needed to understand the impact of structural assumptions. |
| Interventions have constant returns to scale. | Diminishing returns as coverage rises may decrease the value of investments in absolute terms. However, the relative ranking of programs, assuming that marginalized PWID are equally difficult to enroll in any program, would not be affected. Moreover, our framing of low, medium, and high coverage levels is meant to be illustrative of possible investment patterns, but our prioritization rankings and cost-effectiveness conclusions do not change with arbitrary definitions of “high” coverage (S1 Appendix, Section 5.2.3). In fact, we stress that, in practice, achievable coverage levels may vary extensively by region. However, our cost-efficient frontier (Figure 2) suggests that OAT investments along a range of feasible coverage levels can provide high value, even when such levels are below 50%. |
| HCV not explicitly modeled. | We use a high competing mortality risk for PWID to account for both overdose and comorbidities (thus accounting indirectly for HCV comorbidity). OAT and NSP likely have further benefits we have not captured since we do not account for their reduction in the spread of HCV. Nonetheless, since we estimate that both of these programs are cost-effective, implicit bias against these interventions would not undermine our conclusions in this context. Further work is needed to better understand the effects of age, HCV prevalence, and mortality on HIV prevention programs. |

**Figure A: Alternative coverage analysis**


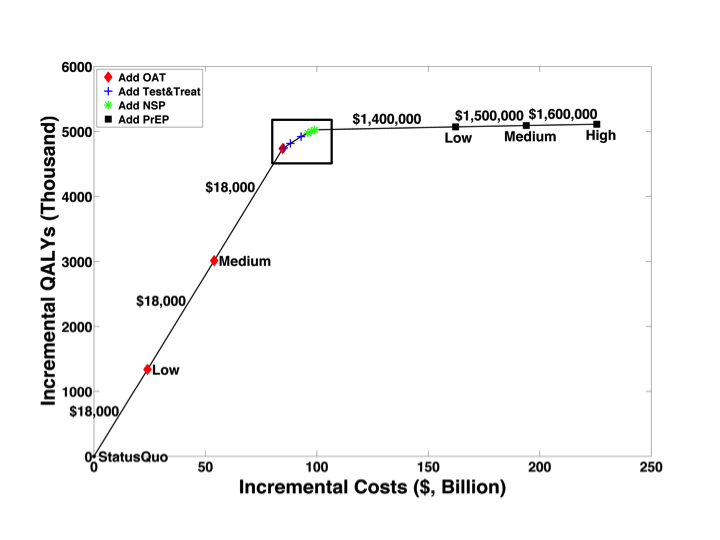

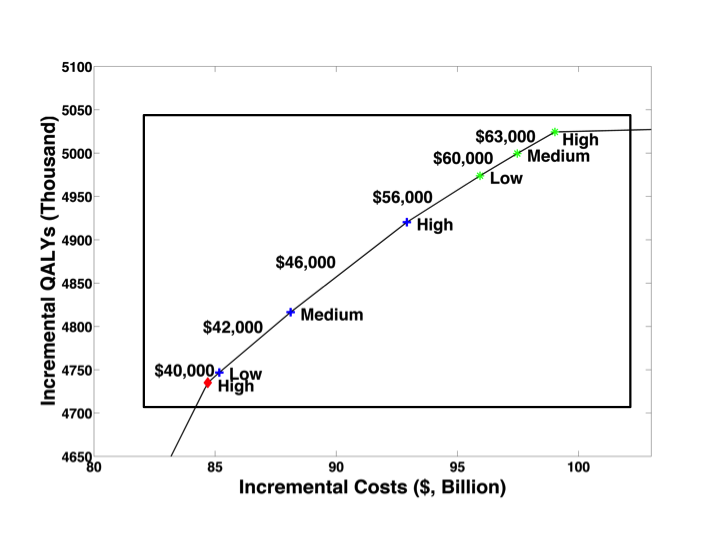


OAT = opioid agonist therapy. NSP = needle-syringe exchange program. PrEP = pre-exposure prophylaxis. QALY = quality-adjusted life year. We considered all possible combinations of programs (OAT, NSP, Test & Treat, and PrEP) at status quo, low (40% coverage), medium (60%), and high coverage (80%) levels) and plotted the resulting cost-efficient frontier with incremental QALYs on the y-axis versus incremental costs on the x-axis. The right panel is an enlargement of the region indicated by the box in the left panel. The figure illustrates that prioritizing expansions of OAT coverage and then investing in Test & Treat and NSP can deliver a high-value portfolio of interventions.

**Figure B: Incremental QALYs versus budget impact, with spending increments of $750 million**


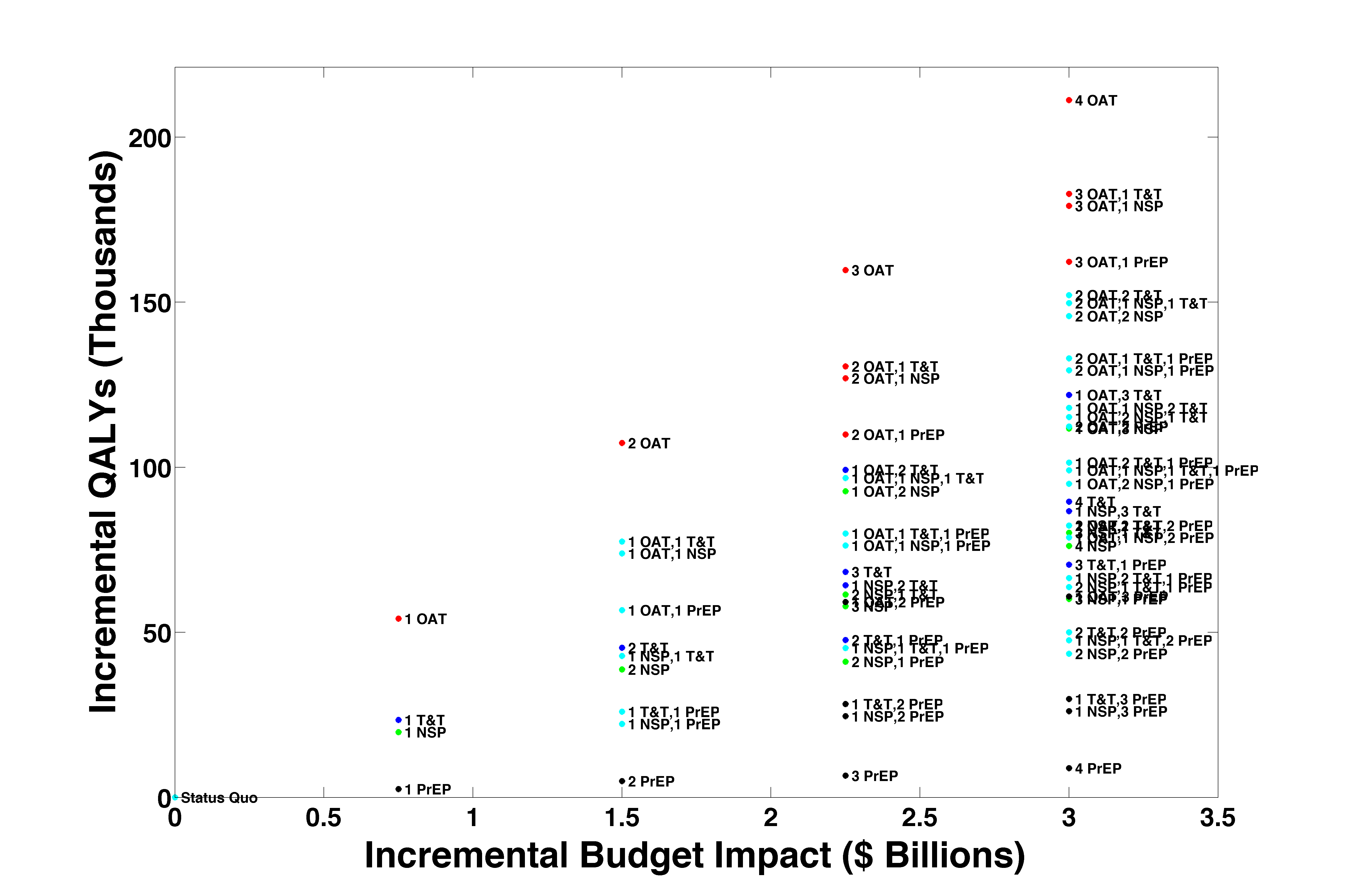


Numbers next to the intervention name correspond to budget units invested in that intervention (in increments of $750 million). OAT = opioid agonist therapy. NSP= needle-syringe exchange program. T&T = Test & Treat. PrEP = pre-exposure prophylaxis. QALY= quality-adjusted life year. Points where investment in OAT exceeds other interventions are red; NSP, green; ART, dark blue; PrEP, black. Where there is no clear “top” intervention, the point is light blue.

**Figure C: HIV infections averted versus budget impact, with spending increments of $750 million**


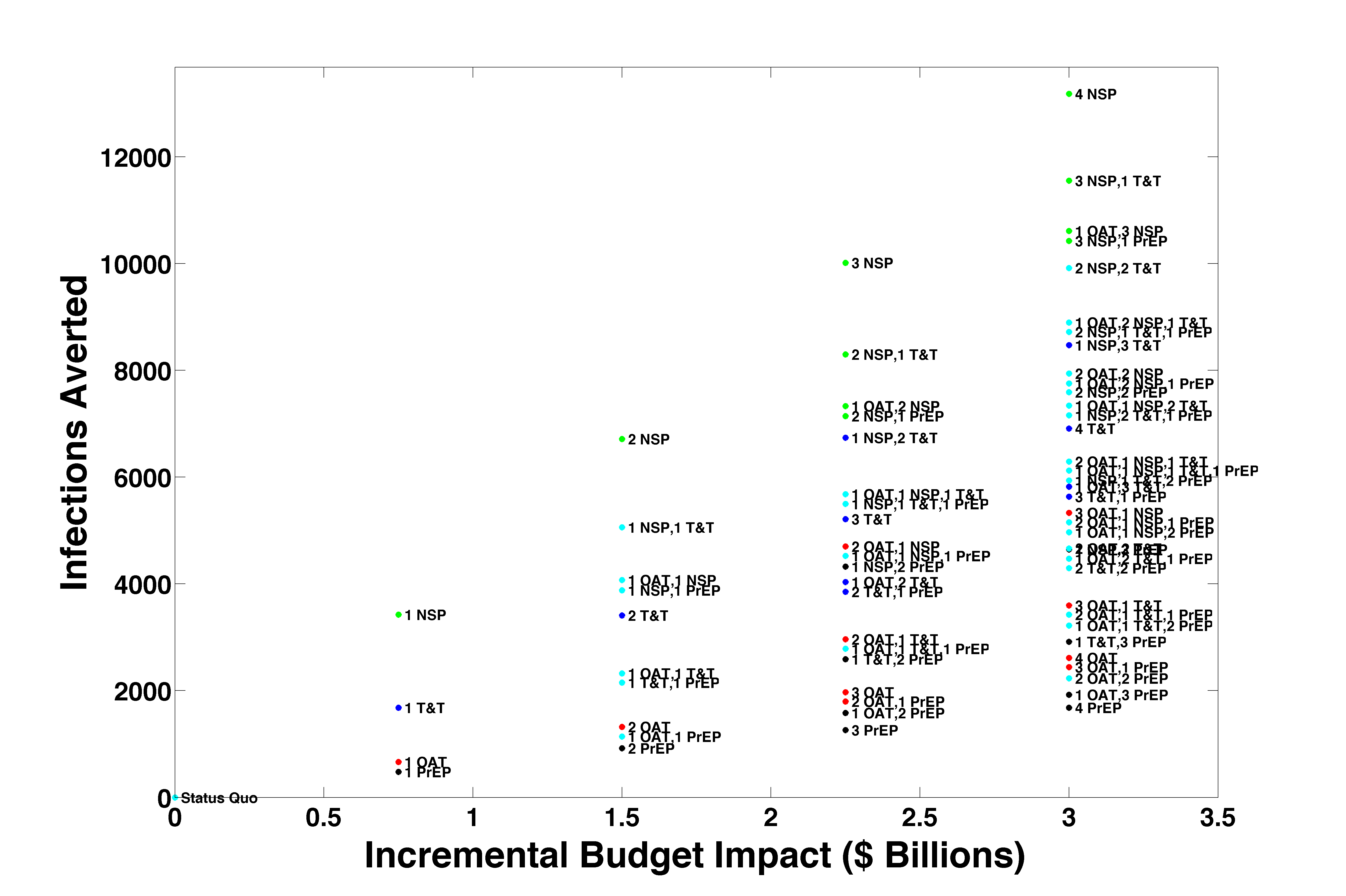


Numbers next to the intervention name correspond to budget units invested in that intervention (in increments of $750 million). OAT = opioid agonist therapy. NSP= needle-syringe exchange program. T&T = Test & Treat. PrEP = pre-exposure prophylaxis. QALY= quality-adjusted life year. Points where investment in OAT exceeds other interventions are red; NSP, green; ART, dark blue; PrEP, black. Where there is no clear “top” intervention, the point is light blue.

**S1 APPENDIX REFERENCES**

1. Bernard CL, Brandeau ML, Owens DK, Humphreys K, Bendavid E, Weyant C, et al. Cost-effectiveness of HIV preexposure prophylaxis for people who inject drugs in the United States. Annals of internal medicine. 2016;165(1):10-9.

2. Arias E. United States life tables, 2010. National vital statistics reports : from the Centers for Disease Control and Prevention, National Center for Health Statistics, National Vital Statistics System. 2014;63(7):1-63. Epub 2014/11/11. PubMed PMID: 25383611.

3. Weinstein MC, O'Brien B, Hornberger J, Jackson J, Johannesson M, McCabe C, et al. Principles of good practice for decision analytic modeling in health-care evaluation: report of the ISPOR Task Force on Good Research Practices--Modeling Studies. Value in health : the journal of the International Society for Pharmacoeconomics and Outcomes Research. 2003;6(1):9-17. Epub 2003/01/22. PubMed PMID: 12535234.

4. Gold M. Panel on cost-effectiveness in health and medicine. Medical care. 1996;34(12 Suppl):Ds197-9. Epub 1996/12/01. PubMed PMID: 8969326.

5. Goldhaber-Fiebert JD, Stout NK, Ortendahl J, Kuntz KM, Goldie SJ, Salomon JA. Modeling human papillomavirus and cervical cancer in the United States for analyses of screening and vaccination. Population health metrics. 2007;5:11. Epub 2007/10/31. doi: 10.1186/1478-7954-5-11. PubMed PMID: 17967185; PubMed Central PMCID: PMCPmc2213637.

6. Karnon J, Vanni T. Calibrating models in economic evaluation: a comparison of alternative measures of goodness of fit, parameter search strategies and convergence criteria. PharmacoEconomics. 2011;29(1):51-62. Epub 2010/12/15. doi: 10.2165/11584610-000000000-00000. PubMed PMID: 21142278.

7. Batina NG, Trentham-Dietz A, Gangnon RE, Sprague BL, Rosenberg MA, Stout NK, et al. Variation in tumor natural history contributes to racial disparities in breast cancer stage at diagnosis. Breast cancer research and treatment. 2013;138(2):519-28. Epub 2013/02/19. doi: 10.1007/s10549-013-2435-z. PubMed PMID: 23417335; PubMed Central PMCID: PMCPmc3610865.

8. Berry DA, Inoue L, Shen Y, Venier J, Cohen D, Bondy M, et al. Modeling the impact of treatment and screening on U.S. breast cancer mortality: a Bayesian approach. Journal of the National Cancer Institute Monographs. 2006;(36):30-6. Epub 2006/10/13. doi: 10.1093/jncimonographs/lgj006. PubMed PMID: 17032892.

9. Stinnett AA, Paltiel AD. Estimating CE ratios under second-order uncertainty: the mean ratio versus the ratio of means. Medical decision making : an international journal of the Society for Medical Decision Making. 1997;17(4):483-9. Epub 1997/10/31. PubMed PMID: 9343807.

10. Cipriano LE, Zaric GS, Holodniy M, Bendavid E, Owens DK, Brandeau ML. Cost effectiveness of screening strategies for early identification of HIV and HCV infection in injection drug users. PloS one. 2012;7(9):e45176. Epub 2012/10/03. doi: 10.1371/journal.pone.0045176. PubMed PMID: 23028828; PubMed Central PMCID: PMCPmc3445468.

11. Zaric GS, Barnett PG, Brandeau ML. HIV transmission and the cost-effectiveness of methadone maintenance. American journal of public health. 2000;90(7):1100-11. Epub 2000/07/18. PubMed PMID: 10897189; PubMed Central PMCID: PMCPmc1446290.

12. Spiller MW, Broz D, Wejnert C, Nerlander L, Paz-Bailey G. HIV infection and HIV-associated behaviors among persons who inject drugs--20 cities, United States, 2012. MMWR Morbidity and mortality weekly report. 2015;64(10):270-5. Epub 2015/03/20. PubMed PMID: 25789742.

13. Broz D, Wejnert C, Pham HT, DiNenno E, Heffelfinger JD, Cribbin M, et al. HIV infection and risk, prevention, and testing behaviors among injecting drug users -- National HIV Behavioral Surveillance System, 20 U.S. cities, 2009. Morbidity and mortality weekly report Surveillance summaries (Washington, DC : 2002). 2014;63(6):1-51. Epub 2014/07/06. PubMed PMID: 24990587.

14. Kral AH, Lorvick J, Ciccarone D, Wenger L, Gee L, Martinez A, et al. HIV prevalence and risk behaviors among men who have sex with men and inject drugs in San Francisco. Journal of urban health : bulletin of the New York Academy of Medicine. 2005;82(1 Suppl 1):i43-50. Epub 2005/03/02. doi: 10.1093/jurban/jti023. PubMed PMID: 15738321; PubMed Central PMCID: PMCPmc3456175.

15. MacArthur GJ, Minozzi S, Martin N, Vickerman P, Deren S, Bruneau J, et al Opiate substitution treatment and HIV transmission in people who inject drugs: systematic review and meta-analysis BMJ 2012;345:e5945. doi: 10.1136/bmj.e5945. PubMed PMID: 23038795; PubMed Central PMCID: PMCPmc3489107.

16. Sullivan LE, Moore BA, Chawarski MC, Pantalon MV, Barry D, O'Connor PG, et al. Buprenorphine/naloxone treatment in primary care is associated with decreased human immunodeficiency virus risk behaviors. Journal of substance abuse treatment. 2008;35(1):87-92. Epub 2007/10/16. doi: 10.1016/j.jsat.2007.08.004. PubMed PMID: 17933486; PubMed Central PMCID: PMCPmc2587397.

17. Degenhardt L, Hall W, Warner-Smith M. Using cohort studies to estimate mortality among injecting drug users that is not attributable to AIDS. Sexually transmitted infections. 2006;82 Suppl 3:iii56-63. Epub 2006/06/01. doi: 10.1136/sti.2005.019273. PubMed PMID: 16735295; PubMed Central PMCID: PMCPmc2576734.

18. Alistar SS, Owens DK, Brandeau ML. Effectiveness and cost effectiveness of oral pre-exposure prophylaxis in a portfolio of prevention programs for injection drug users in mixed HIV epidemics. PloS one. 2014;9(1):e86584. Epub 2014/02/04. doi: 10.1371/journal.pone.0086584. PubMed PMID: 24489747; PubMed Central PMCID: PMCPmc3904940.

19. Pollack HA, D'Aunno T. HIV testing and counseling in the nation's outpatient substance abuse treatment system, 1995-2005. Journal of substance abuse treatment. 2010;38(4):307-16. Epub 2010/02/23. doi: 10.1016/j.jsat.2009.12.004. PubMed PMID: 20171038.

20. Sanchez T, Finlayson T, Drake A, Behel S, Cribbin M, Dinenno E, et al. Human immunodeficiency virus (HIV) risk, prevention, and testing behaviors--United States, National HIV Behavioral Surveillance System: men who have sex with men, November 2003-April 2005. Morbidity and mortality weekly report Surveillance summaries (Washington, DC : 2002). 2006;55(6):1-16. Epub 2006/07/11. PubMed PMID: 16826162.

21. McCollister KE, French MT, Pyne JM, Booth B, Rapp R, Carr C The cost of treating addiction from the client’s perspective: Results from a multi-modality application of the Client DATCAP Drug Alcohol Depend 2009;104(3):241-248.

22. Des Jarlais DC, Nugent A, Solberg A, Feelemyer J, Mermin J, Holtzman D. Syringe service programs for persons who inject drugs in urban, suburban, and rural areas - United States, 2013. MMWR Morbidity and mortality weekly report. 2015;64(48):1337-41. Epub 2015/12/15. doi: 10.15585/mmwr.mm6448a3. PubMed PMID: 26655918.

23. Strathdee SA, Beyrer C. Threading the needle--how to stop the HIV outbreak in rural Indiana. The New England journal of medicine. 2015;373(5):397-9. Epub 2015/06/25. doi: 10.1056/NEJMp1507252. PubMed PMID: 26106947.

24. Burt RD, Thiede H. Reduction in needle sharing among Seattle-area injection drug users across 4 surveys, 1994-2013. American journal of public health. 2016;106(2):301-7. Epub 2015/12/23. doi: 10.2105/ajph.2015.302959. PubMed PMID: 26691117.

25. Kaplan EH, Heimer R. A model-based estimate of HIV infectivity via needle sharing. Journal of acquired immune deficiency syndromes (1999). 1992;5(11):1116-8. Epub 1992/01/01. PubMed PMID: 1403641.

26. Holtgrave DR, Pinkerton SD, Jones TS, Lurie P, Vlahov D. Cost and cost-effectiveness of increasing access to sterile syringes and needles as an HIV prevention intervention in the United States. Journal of acquired immune deficiency syndromes and human retrovirology : official publication of the International Retrovirology Association. 1998;18 Suppl 1:S133-8. Epub 1998/07/15. PubMed PMID: 9663636.

27. Knittel S Bill seeks to end the ban on syringe exchange programs Seattle Gay News 17 July 2009 Accessed at <http://wwwsgnorg/sgnnews37_29/page14cfm> on January 22, 2016.

28. Kim NJ, Jin H, McFarland W, Raymond HF. Trends in sources and sharing of needles among people who inject drugs, San Francisco, 2005-2012. The International journal on drug policy. 2015;26(12):1238-43. Epub 2015/09/15. doi: 10.1016/j.drugpo.2015.08.013. PubMed PMID: 26365768.

29. Centers for Disease Control and Prevention HIV/AIDS: Infographics and Posters Accessed at wwwcdcgov/hiv/library/infographicshtml?s_cid=bb-dhap-hiv-ngmhaad-001–ngmhaad on June 29, 2015.

30. Spire B, Lucas GM, Carrieri MP. Adherence to HIV treatment among IDUs and the role of opioid substitution treatment (OST). The International journal on drug policy. 2007;18(4):262-70. Epub 2007/08/11. doi: 10.1016/j.drugpo.2006.12.014. PubMed PMID: 17689374.

31. Westergaard RP, Ambrose BK, Mehta SH, Kirk GD. Provider and clinic-level correlates of deferring antiretroviral therapy for people who inject drugs: a survey of North American HIV providers. Journal of the International AIDS Society. 2012;15(1):10. Epub 2012/03/01. doi: 10.1186/1758-2652-15-10. PubMed PMID: 22360788; PubMed Central PMCID: PMCPmc3306203.

32. Skarbinski J, Rosenberg E, Paz-Bailey G, Hall HI, Rose CE, Viall AH, et al. Human immunodeficiency virus transmission at each step of the care continuum in the United States. JAMA internal medicine. 2015;175(4):588-96. Epub 2015/02/24. doi: 10.1001/jamainternmed.2014.8180. PubMed PMID: 25706928.

33. Centers for Disease Control and Prevention Understanding the HIV Care Continuum [cited 2016 Mar]; Accessed at: <http://wwwcdcgov/hiv/pdf/DHAP_Continuumpdf>.

34. Long EF, Brandeau ML, Owens DK. The cost-effectiveness and population outcomes of expanded HIV screening and antiretroviral treatment in the United States. Annals of internal medicine. 2010;153(12):778-89. Epub 2010/12/22. doi: 10.7326/0003-4819-153-12-201012210-00004. PubMed PMID: 21173412; PubMed Central PMCID: PMCPmc3173812.

35. Kapadia F, Latka MH, Hudson SM, Golub ET, Campbell JV, Bailey S, et al. Correlates of consistent condom use with main partners by partnership patterns among young adult male injection drug users from five US cities. Drug and alcohol dependence. 2007;91 Suppl 1:S56-63. Epub 2007/03/03. doi: 10.1016/j.drugalcdep.2007.01.004. PubMed PMID: 17329041.

36. Hollingsworth TD, Anderson RM, Fraser C. HIV-1 transmission, by stage of infection. The Journal of infectious diseases. 2008;198(5):687-93. Epub 2008/07/30. doi: 10.1086/590501. PubMed PMID: 18662132.

37. Samji H, Cescon A, Hogg RS, Modur SP, Althoff KN, Buchacz K, et al. Closing the gap: increases in life expectancy among treated HIV-positive individuals in the United States and Canada. PloS one. 2013;8(12):e81355. Epub 2013/12/25. doi: 10.1371/journal.pone.0081355. PubMed PMID: 24367482; PubMed Central PMCID: PMCPmc3867319.

38. Wada N, Jacobson LP, Cohen M, French A, Phair J, Munoz A. Cause-specific mortality among HIV-infected individuals, by CD4(+) cell count at HAART initiation, compared with HIV-uninfected individuals. AIDS (London, England). 2014;28(2):257-65. Epub 2013/10/10. doi: 10.1097/qad.0000000000000078. PubMed PMID: 24105030; PubMed Central PMCID: PMCPmc4164055.

39. Castilla J, Del Romero J, Hernando V, Marincovich B, Garcia S, Rodriguez C. Effectiveness of highly active antiretroviral therapy in reducing heterosexual transmission of HIV. Journal of acquired immune deficiency syndromes (1999). 2005;40(1):96-101. Epub 2005/08/27. PubMed PMID: 16123689.

40. Choopanya K, Martin M, Suntharasamai P, Sangkum U, Mock PA, Leethochawalit M, et al. Antiretroviral prophylaxis for HIV infection in injecting drug users in Bangkok, Thailand (the Bangkok Tenofovir Study): a randomised, double-blind, placebo-controlled phase 3 trial. Lancet. 2013;381(9883):2083-90. Epub 2013/06/19. doi: 10.1016/s0140-6736(13)61127-7. PubMed PMID: 23769234.

41. Sanders GD, Bayoumi AM, Sundaram V, Bilir SP, Neukermans CP, Rydzak CE, et al. Cost-effectiveness of screening for HIV in the era of highly active antiretroviral therapy. The New England journal of medicine. 2005;352(6):570-85. Epub 2005/02/11. doi: 10.1056/NEJMsa042657. PubMed PMID: 15703422.

42. Juusola JL, Brandeau ML, Owens DK, Bendavid E. The cost-effectiveness of preexposure prophylaxis for HIV prevention in the United States in men who have sex with men. Annals of internal medicine. 2012;156(8):541-50. Epub 2012/04/18. doi: 10.7326/0003-4819-156-8-201204170-00001. PubMed PMID: 22508731; PubMed Central PMCID: PMCPmc3690921.

43. Law Enforcement Assisted Diversion (LEAD) About LEAD Accessed at leadkingcountyorg/ on March 15, 2016.

44. Barnett PG, Chow A, Joyce VR, Bayoumi AM, Griffin SC, Sun H, et al. Effect of management strategies and clinical status on costs of care for advanced HIV. The American journal of managed care. 2014;20(5):e129-37. Epub 2014/10/21. PubMed PMID: 25326927.

45. Paltiel AD, Weinstein MC, Kimmel AD, Seage GR, 3rd, Losina E, Zhang H, et al. Expanded screening for HIV in the United States--an analysis of cost-effectiveness. The New England journal of medicine. 2005;352(6):586-95. Epub 2005/02/11. doi: 10.1056/NEJMsa042088. PubMed PMID: 15703423.

46. Centers for Disease Control and Prevention, U.S. Public Health Service. Preexposure prophylaxis for the prevention of HIV infection in the United States - 2014: A Clinical Practice Guideline. 2014. Accessed at <http://www.cdc.gov/hiv/pdf/prepguidelines2014.pdf> on June 12, 2014.

47. New York State Department of Health Pre-exposure prophylaxis (PrEP) to prevent HIV infection: questions and answers December 2012 Accessed at wwwhealthnygov/publications/0265/ on February 9, 2015.

48. Paltiel AD, Freedberg KA, Scott CA, Schackman BR, Losina E, Wang B, et al. HIV preexposure prophylaxis in the United States: impact on lifetime infection risk, clinical outcomes, and cost-effectiveness. Clinical infectious diseases : an official publication of the Infectious Diseases Society of America. 2009;48(6):806-15. Epub 2009/02/06. doi: 10.1086/597095. PubMed PMID: 19193111; PubMed Central PMCID: PMCPmc2876329.

49. US Department of Veterans Affairs National Acquisition Center (CCST) Accessed at www1vagov/nac/ on April 1, 2015.

50. Friedman SR, Tempalski B, Cooper H, Perlis T, Keem M, Friedman R, et al. Estimating numbers of injecting drug users in metropolitan areas for structural analyses of community vulnerability and for assessing relative degrees of service provision for injecting drug users. Journal of urban health : bulletin of the New York Academy of Medicine. 2004;81(3):377-400. Epub 2004/07/27. doi: 10.1093/jurban/jth125. PubMed PMID: 15273263; PubMed Central PMCID: PMCPmc3455936.

51. Tempalski B, Pouget ER, Cleland CM, Brady JE, Cooper HL, Hall HI, et al. Trends in the population prevalence of people who inject drugs in US metropolitan areas 1992-2007. PloS one. 2013;8(6):e64789. Epub 2013/06/12. doi: 10.1371/journal.pone.0064789. PubMed PMID: 23755143; PubMed Central PMCID: PMCPmc3673953.

52. Integrated prevention services for HIV infection, viral hepatitis, sexually transmitted diseases, and tuberculosis for persons who use drugs illicitly: summary guidance from CDC and the U.S. Department of Health and Human Services. MMWR Recommendations and reports : Morbidity and mortality weekly report Recommendations and reports / Centers for Disease Control. 2012;61(Rr-5):1-40. Epub 2012/11/09. PubMed PMID: 23135062.

53. Pollack HA, D'Aunno T. Dosage patterns in methadone treatment: results from a national survey, 1988-2005. Health services research. 2008;43(6):2143-63. Epub 2008/06/05. doi: 10.1111/j.1475-6773.2008.00870.x. PubMed PMID: 18522665; PubMed Central PMCID: PMCPMC2613988.

54. Duintjer Tebbens RJ, Thompson KM, Hunink MG, Mazzuchi TA, Lewandowski D, Kurowicka D, et al. Uncertainty and sensitivity analyses of a dynamic economic evaluation model for vaccination programs. Medical decision making : an international journal of the Society for Medical Decision Making. 2008;28(2):182-200. Epub 2008/03/20. doi: 10.1177/0272989x07311752. PubMed PMID: 18349438.

55. Bellis DJ. Reduction of AIDS risk among 41 heroin addicted female street prostitutes: effects of free methadone maintenance. Journal of addictive diseases. 1993;12(1):7-23. Epub 1993/01/01. doi: 10.1300/J069v12n01_02. PubMed PMID: 8381030.

1. This column shows the optimal portfolio construction as informed by the combinations of interventions falling sequentially on the cost-efficient frontier. Each combination is represented as a 4-digit number in brackets. Each digit references the type of intervention, [OAT NSP T&T PrEP], and its value corresponds to the coverage level (1=low, 2=medium, 3=high) for that intervention. [↑](#footnote-ref-1)
2. ICER = Incremental cost-effectiveness ratio. The listed value refers to the ICER the first time the intervention is added to a portfolio falling on the cost-efficient frontier. [↑](#footnote-ref-2)
3. Incremental costs, incremental QALYs, and the ICER of each portfolio are relative to the next best intervention. OAT = opioid agonist therapy. NSP = needle-syringe exchange program. PrEP = pre-exposure prophylaxis. PWID = people who inject drugs. QALY = quality-adjusted life year. ICER = incremental cost-effectiveness ratio. [↑](#footnote-ref-3)
4. Distributions on calibrated model parameters can be found in the supplement of a previously published paper (7). [↑](#footnote-ref-4)
5. We use Beta, Gamma, and (truncated) log-normal distributions to describe the uncertainty around model parameters. A Beta(a, b) distribution has mean a/(a+b) and variance (ab)/[(a+b+1)(a+b)^2^]. A Gamma(a,b) distribution has mean ab and variance ab^2^. A log-normal, logN(μ,σ), distribution has mean exp(μ+σ^2^/2) and variance exp(σ^2^-1)exp(2μ+σ^2^). We truncate the log-normal distribution if it goes outside the feasible range of the model parameter (e.g., a hazard ratio that decreases risk should not exceed 1, while a hazard ratio that increases risk should not drop below 1). [↑](#footnote-ref-5)
6. Percent of PSA samples for which the program is the priority investment. OAT = opioid agonist therapy. NSP = needle-syringe exchange program. PrEP = pre-exposure prophylaxis. [↑](#footnote-ref-6)
7. Percent of PSA samples for which the addition of each program to the optimal portfolio/cost-efficient frontier costs less than the specified threshold. [↑](#footnote-ref-7)
8. For joint sensitivity analysis, 3 calibrated sets, each sampled 30 times, have been selected so that OAT minimally decreases injection frequency and awareness does not affect injecting equipment sharing frequency. S1 Appendix, Section 5.3 provides details. [↑](#footnote-ref-8)
9. For joint sensitivity analysis, calibrated sets have been altered so that OAT does not decrease injection frequency and awareness does not affect injecting equipment sharing frequency. S1 Appendix, Section 5.3 provides details. [↑](#footnote-ref-9)
